# *ARID1B*-related disorder in 87 adults: Natural history and self-sustainability

**DOI:** 10.1016/j.gimo.2024.101873

**Published:** 2024-07-23

**Authors:** P.J. van der Sluijs, M. Gösgens, A.J.M. Dingemans, P. Striano, A. Riva, C. Mignot, A. Faudet, G. Vasileiou, M. Walther, S.A. Schrier Vergano, M. Alders, F.S. Alkuraya, I. Alorainy, H.S. Alsaif, B. Anderlid, I. Bache, I. van Beek, M. Blanluet, B.W. van Bon, T. Brunet, H. Brunner, M.L. Carriero, P. Charles, N. Chatron, E. Coccia, C. Dubourg, R.K. Earl, E.E. Eichler, L. Faivre, N. Foulds, C. Graziano, A.M. Guerrot, M.O. Hashem, S. Heide, D. Heron, S.E. Hickey, S.M.J. Hopman, A. Kattentidt-Mouravieva, J. Kerkhof, J.S. Klein Wassink-Ruiter, E.C. Kurtz-Nelson, K. Kušíková, M. Kvarnung, F. Lecoquierre, G.S. Leszinski, L. Loberti, P.L. Magoulas, F. Mari, I. Maystadt, G. Merla, J.M. Milunsky, S. Moortgat, G. Nicolas, M.O.’ Leary, S. Odent, J.R. Ozmore, K. Parbhoo, R. Pfundt, M. Piccione, A.M. Pinto, B. Popp, A. Putoux, H.L. Rehm, A. Reis, A. Renieri, J.A. Rosenfeld, M. Rossi, E. Salzano, P. Saugier-Veber, M. Seri, G. Severi, F.M. Sonmez, G. Strobl-Wildemann, K.E. Stuurman, E. Uctepe, H. Van Esch, G. Vitetta, B.B.A. de Vries, D. Wahl, T. Wang, P. Zacher, K.R. Heitink, F.G. Ropers, D. Steenbeek, T. Rybak, G.W.E. Santen

**Affiliations:** 1 Department of Clinical Genetics, Leiden University Medical Center, Leiden, the Netherlands; 2 Department of Human Genetics, Donders Institute for Brain, Cognition and Behaviour, Radboud University Medical Center, Nijmegen, the Netherlands; 3 Pediatric Neurology and Muscular Diseases Unit, IRCCS Istituto Giannina Gaslini, Genova, Italy; 4 Department of Neurosciences, Rehabilitation, Ophthalmology, Genetics, Maternal and Child Health, University of Genova, Genova, Italy; 5 IRCCS Istituto Giannina Gaslini, Genova, Italy; 6 Service de génétique médicale, APHP Pitié-Salpêtrière, Paris, France; 7 Département de Génétique, Assistance publique - Hôpitaux de Paris Sorbonne Université, Hôpital Pitié-Salpêtrière et Trousseau, Paris, France; 8 Institute of Human Genetics, Universitätsklinikum Erlangen, Friedrich-Alexander-Universität Erlangen-Nürnberg, 91054 Erlangen, Germany; 9 Centre for Rare Diseases Erlangen (ZSEER), Erlangen, Germany; 10 Children's Hospital of The King's Daughters, Norfolk, VA; 11 Department of Pediatrics, Eastern Virginia Medical School, Norfolk, VA; 12 Section Clinical Genetics, Department of Human Genetics, Amsterdam University Medical Centers, Amsterdam, the Netherlands; 13 Department of Translational Genomics, Center for Genomic Medicine, King Faisal Specialist Hospital and Research Center, Riyadh, Saudi Arabia; 14 Department of Anatomy and Cell Biology, College of Medicine, Alfaisal University, Riyadh, Saudi Arabia; 15 Department of Radiology and Diagnostic Imaging, King Khalid University Hospital and College of Medicine, King Saud University, Riyadh, Saudi Arabia; 16 Centre of Excellence for Biomedicine, Joint Centers of Excellence Program, King Abdulaziz City for Science and Technology, Riyadh, Saudi Arabia; 17 Clinical Genetics Karolinska Universitet Hospital and Department of Molecular Medicine and Surgery, Karolinska Institutet, Stockholm, Sweden; 18 Department of Clinical Genetics, Copenhagen University Hospital, Rigshospitalet, Copenhagen, Denmark; 19 Service de Génétique Oncologique, Institut Curie, Paris, France; 20 Department of Human Genetics, Radboud University Medical Center, Nijmegen, the Netherlands; 21 Institute of Human Genetics, Klinikum rechts der Isar, School of Medicine, Technical University of Munich, Munich, Germany; 22 Department of Pediatric Neurology and Developmental Medicine and LMU Center for Children with Medical Complexity, Dr. von Hauner Children's Hospital, LMU Hospital, Ludwig-Maximilians-University, D-80337 Munich, Germany; 23 Medical Genetics, University of Siena, Siena, Italy; 24 Service de génétique, Hospices Civils de Lyon ERN ITHACA, INSERM U1028, CNRS UMR5292, Centre de Recherche en Neurosciences de Lyon, GENDEV Team, Université Claude Bernard Lyon 1, Bron, France; 25 Institut Neuromyogène, Laboratoire Physiopathologie et Génétique du Neurone et du Muscle, Equipe Métabolisme énergétique et développement neuronal, CNRS UMR 5310, INSERM U1217, Université Lyon 1, Lyon, France; 26 Department of Medical and Surgical Science, Postgraduate School of Medical Genetics, Alma Mater Studiorum University of Bologna, Bologna, Italy; 27 Service de Génétique Moléculaire et Génomique Médicale, CHU de Rennes, Rennes, France; 28 Univ Rennes, CNRS, INSERM, IGDR (Institut de génétique et développement de Rennes) - UMR 6290, ERL U1305, RENNES, France; 29 Department of Psychiatry and Behavioral Sciences, University of Washington, Seattle, WA; 30 Department of Genome Sciences, University of Washington School of Medicine, Seattle, WA; 31 Howard Hughes Medical Institute, University of Washington, School of Medicine, Seattle, WA; 32 Centre de Référence Anomalies du Développement et Syndromes Malformatifs, FHU TRANSLAD, CHU Dijon, Dijon, France; 33 Genetics of Developmental Disorders, INSERM - Bourgogne Franche-Comté University, UMR 1231 GAD Team, Dijon, France; 34 Wessex Clinical Genetics Service, University Hospital Southampton, Princess Anne Hospital, Southampton, United Kingdom; 35 Medical Genetics Unit, AUSL Romagna, Cesena, Italy; 36 Department of Genetics and reference Center for Developmental Disorders, Univ Rouen Normandie, Normandie Univ, Inserm U1245 and CHU Rouen, Rouen, France; 37 Division of Genetic & Genomic Medicine, Nationwide Children's Hospital, Columbus, OH; 38 Department of Pediatrics, The Ohio State University College of Medicine, Columbus, OH; 39 Department of Genetics, University Medical Center Utrecht, Utrecht, the Netherlands; 40 Stichting Zuidwester, Middelharnis, the Netherlands; 41 Verspeeten Clinical Genome Centre, London Health Sciences Centre, London, ON, Canada; 42 Department of Genetics, University of Groningen, University Medical Center Groningen, the Netherlands; 43 Department of Pediatrics, Indiana University School of Medicine, Indianapolis, IN; 44 Department of Pediatric Neurology, Faculty of Medicine, Comenius University and National Institute of Children's Diseases, Bratislava, Slovakia; 45 Department of Medical Biotechnologies, Med Biotech Hub and Competence Centre, University of Siena, Siena, Italy; 46 Department of Molecular and Human Genetics, Baylor College of Medicine, Houston, TX; 47 Centre de Génétique Humaine, Institut de Pathologie et de Génétique, Gosselies, Belgium; 48 Department of Molecular Medicine & Medical Biotechnology, University of Naples Federico II, Naples, Italy; 49 Laboratory of Regulatory & Functional Genomics, Fondazione IRCCS Casa Sollievo della Sofferenza, San Giovanni Rotondo, Foggia, Italy; 50 Center for Human Genetics Inc, Cambridge, MA; 51 Broad Center for Mendelian Genomics, Broad Institute of MIT and Harvard, Cambridge, MA; 52 Centre de Référence Maladies Rares CLAD-Ouest, ERN-ITHACA, FHU GenOMedS, CHU de Rennes, RENNES, France; 53 Medical Genetics, Dartmouth Hitchcock Medical Center, Lebanon, NH; 54 The Steve and Cindy Rasmussen Institute for Genomic Medicine at Nationwide Children's Hospital, Columbus, OH; 55 Medical Genetics Unit, AOOR Villa Sofia-Cervello Hospitals, Palermo, Italy; 56 Department of Health Promotion, Mother and Child Care, Internal Medicine and Medical Specialties, University of Palermo, Palermo, Italy; 57 Berlin Institute of Health at Charitè, Universitätsklinikum Berlin, Centre of Functional Genomics, Berlin, Germany; 58 Baylor Genetics Laboratories, Houston, TX; 59 Department of Child Neurology, Karadeniz Technical University Faculty of Medicine, Retired Lecturer, Trabzon, Turkey; 60 MVZ Humangenetik Ulm, Ulm, Germany; 61 Department of Clinical Genetics, Erasmus MC, University Medical Center Rotterdam, Rotterdam, the Netherlands; 62 Acıbadem Labmed Ankara Tissue Typing Laboratory, Ankara, Turkey; 63 Center for Human Genetics, University Hospitals Leuven, Leuven, Belgium; 64 Department of Clinical Genetics, MVZ Martinsried, Munich, Germany; 65 Department of Medical Genetics, Center for Medical Genetics, School of Basic Medical Sciences, Peking University, Beijing, China; 66 Neuroscience Research Institute, Peking University Key Laboratory for Neuroscience, Ministry of Education of China & National Health Commission of China, Beijing, China; 67 Autism Research Center, Peking University Health Science Center, Beijing, China; 68 Epilepsy Center Kleinwachau, Radeberg, Germany; 69 Department of Rehabilitation Medicine, Leiden University Medical Center, Leiden, the Netherlands; 70 Willem-Alexander Children’s Hospital, department of Pediatrics, Leiden University Medical Center, the Netherlands; 71 Department of Rehabilitation Medicine, Maastricht University Medical Center / Adelante Rehabilitation, Maastricht, The Netherlands; 72 's Heeren Loo Noordwijk, Noordwijk, the Netherlands

**Keywords:** Adult, *ARID1B*, Coffin–Siris syndrome, Developmental delay, Intellectual disability

## Abstract

**Purpose:**

*ARID1B* is one of the most frequently mutated genes in intellectual disability cohorts. Thus, far few adult-aged patients with *ARID1B*-related disorder have been described, which limits our understanding of the disease’s natural history and our ability to counsel patients and their families.

**Methods:**

Data on patients aged 18+ years with *ARID1B*-related disorder were collected through an online questionnaire completed by clinicians and parents.

**Results:**

Eighty-seven adult patients with *ARID1B* were included. Cognitive functioning ranged from borderline to severe intellectual disability. Patients identified through the genetic workup of their child were either mosaic or had a variant in exon 1. New clinical features identified in this population are loss of skill (16/64, 25%) and recurrent patella luxation (12/45, 32%). Self-sustainability data showed that 88% (45/51) could eat independently, and 16% (7/45) could travel alone by public transport. Facial photo analysis showed that patients’ photographs taken at different ages clustered consistently, separate from matched controls.

**Conclusion:**

The *ARID1B* spectrum is broad, and as patients age, there is a significant shift in the medical aspects requiring attention. To address the changing medical needs with increasing age, we have formulated recommendations to promote timely intervention in an attempt to mitigate disease progression.

## Introduction

*ARID1B* (HUGO Gene Nomenclature Committee: 18040) is one of the most frequently mutated genes in intellectual disability (ID) and neurodevelopmental delay (NDD) cohorts at around 1%.^1^, ^2^, ^3^, ^4^ The phenotypic spectrum of *ARID1B*-related disorder is broad, ranging from severe ID in Coffin–Siris syndrome (CSS) patients (OMIM 135900) to normal IQ scores in patients with developmental delay.^5^

Although many challenges faced by patients during childhood are well-documented (such as feeding difficulties, failure to thrive, and seizures)^5^, ^6^, ^7^, ^8^ there remains a significant knowledge gap regarding the obstacles they encounter later in adult life. This knowledge gap is common in genetic ID and NDD, as most published patients are minors.

Although there are sporadic case reports of adult patients with *ARID1B*-related disorder,^7^, ^8^, ^9^, ^10^, ^11^ it is unclear whether these represent a biased subset of the phenotype. Thus, studies documenting the development, functioning, and challenges faced by adult-aged patients in large cohorts are needed to improve the counseling of parents about the diagnosis, provide appropriate screening and guidance to diagnosed patients, and facilitate the transition of patients from pediatric to adult care.^12^ To provide a more complete overview of adult patients with *ARID1B*-related disorder, we acquired and analyzed data from 87 adult-aged patients, investigated the natural history of this disorder, assessed functional (in)dependence, identified potential comorbidities associated with aging, and aggregated these data into screening recommendations.

## Materials and Methods

### Patient collection

Patients with a heterozygous pathogenic variant in *ARID1B* and aged 18 years and above were identified through the following diverse sources: national and international colleagues approaching us with their patients; ClinVar; Leiden Open Variant Database; contacts from the Baylor Genetics Laboratories; physician referrals for second opinions; and the Facebook group named ”Coffin-Siris Syndrome Group”. Additionally, recruitment took place through the outpatient CSS expertise center at Leiden University Medical Center, Leiden, the Netherlands.

### Data collection

Data were collected through an online questionnaire which was completed by the referring physician. If possible, parents were also asked to complete an online questionnaire (languages available were English, Dutch, French, German, Italian and Japanese). The clinical questionnaire focused on medical history and physical examination. Although several questions overlapped with those in the parental questionnaire, the parental questionnaire also included additional inquiries about activities of daily living and overall functioning. Variable labels of all questions are included in Supplemental Excel 1. When 2 patients with the same pathogenic variant were included, their phenotypes were compared. If overlapping features were observed, contributing clinicians were contacted to verify whether these were different patients. In this manner, we were able to identify 2 duplicate entries.

### Data assessment

Alamut Visual Plus version 1.6.1 was used to translate the genetic variant or derive the missing genomic location if a genetic variant was reported on a different *ARID1B* transcript, if another genomic build was used, or if the genomic location was not reported. If the standard deviation score (SDS) was not reported, and raw data on weight, height, or occipital–frontal circumference (OFC) were available, the SDS was determined using published growth charts.^13^^,^^14^ Furthermore, we contacted clinicians of published patients with *ARID1B*-related disorders (now aged 18+) by email to request an update. All analyses were executed using SPSS version 25. R version 4.2.1 was used to create graphs. Control data for survival were derived from the Human Mortality database as reported by Beltrán-Sánchez et al^15^ and Verguet et al^16^, data on developmental milestones were derived from Sheldrick et al^17^ and data on toilet training from Schum et al.^18^

### PhenoScore

To assess whether the individuals in this study had a facial gestalt distinguishable from other NDD patients, the facial module of PhenoScore was utilized. PhenoScore is a next-generation phenomics framework that uses artificial intelligence to assess whether the phenotype of a specific group is different from that of age-, sex- and ethnicity-matched controls with neurodevelopmental disorders.^19^ In this case, we used QMagFace^20^ as the facial feature extraction method in PhenoScore and then trained it to see whether the facial features of the investigated individuals were different from those of matched controls with NDD. Matched NDD controls were derived from the in-house database of the Radboud University Medical Center with over 1,200 individuals seen at their outpatient clinic.^19^ The individuals included in this study comprise a sample of patients with Neurodevelopmental Disorders (NDD) seen at the Radboud University Medical Center. This cohort encompasses both individuals with known genetic causes of NDD and those with unknown causes. Most of the individuals in this group have undergone exome sequencing to identify potential genetic factors contributing to their condition. This sample is not biased or overrepresented by specific genetic disorders; rather, it represents a random and non-selected subset of the NDD population. In other words, it reflects the diversity and heterogeneity typically observed within the NDD population. For the full methodology of PhenoScore, please see Dingemans et al.^19^ These analyzes were performed for the whole study group and different subgroups based on age.

## Results

**Figure 1:**
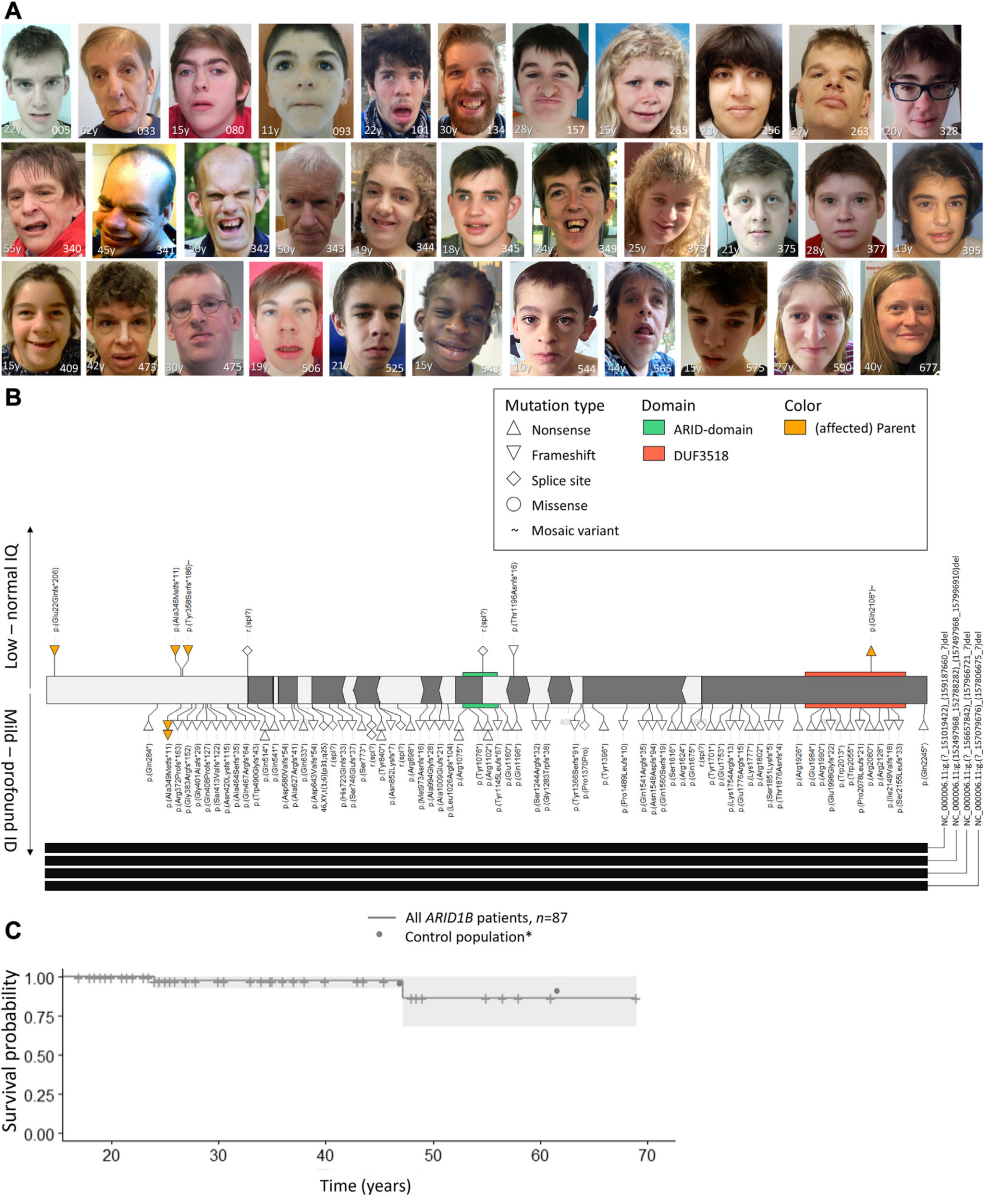

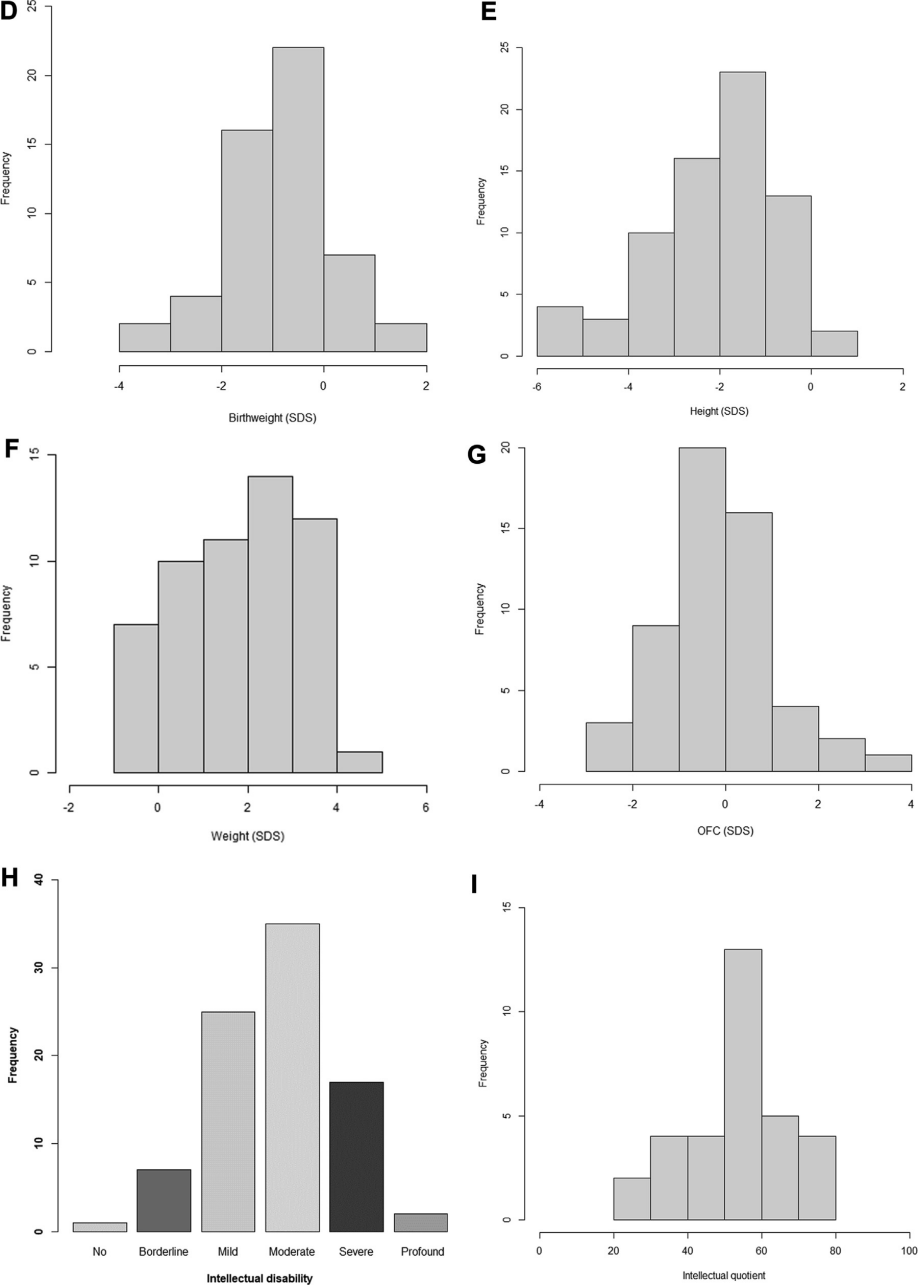

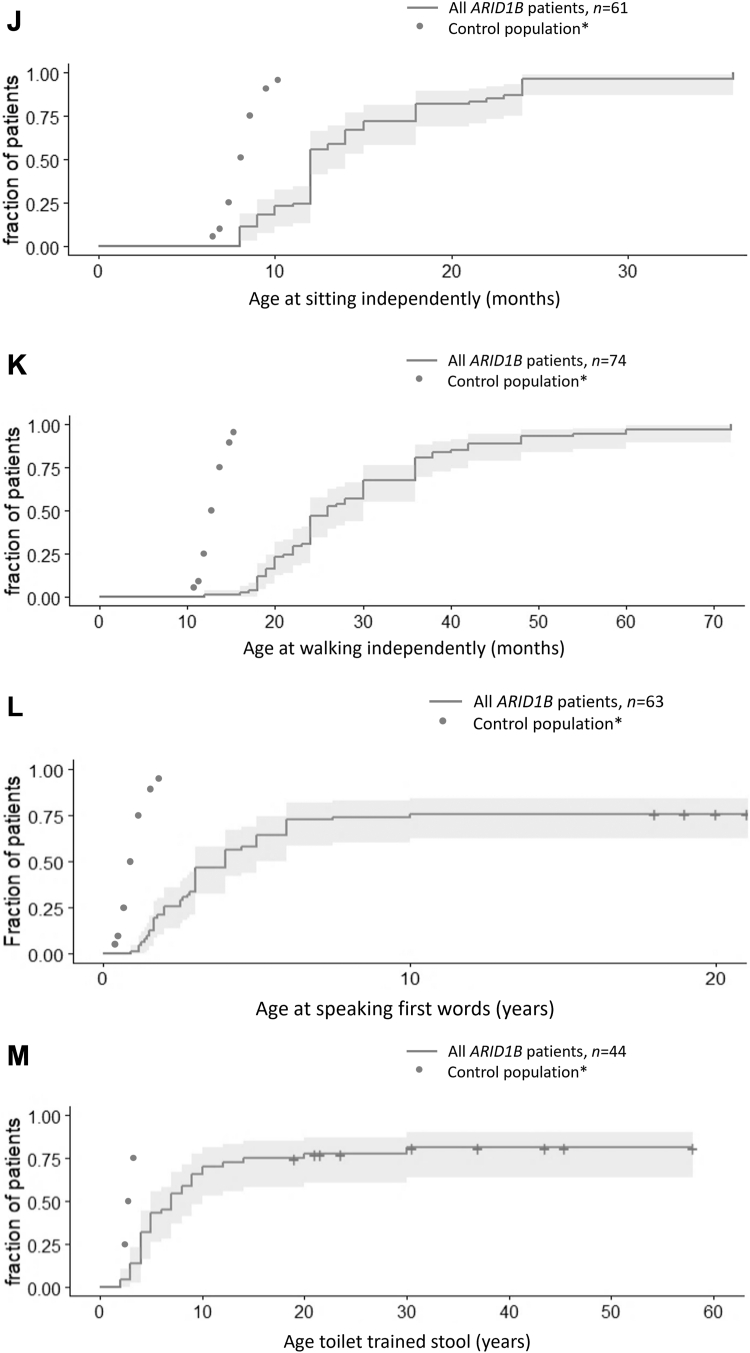
**General characteristicts of the adult*****ARID1B*****population.** A. Facial photos of *ARID1B* patients. B. *ARID1B* transcript with pathogenic variants identified in our patients. C. Survival plot for death *n* = 87. Histogram of the SDS of (D) Birthweight, *n* = 53; (E) Height, *n* = 69; (F) weight for length, *n* = 55; (G) OFC, *n* = 55. H. Distribution of ID severity *n* = 87. I. IQ score *n* = 32. Cumulative distribution of developmental milestones (J) sitting, *n* = 61; (K) walking, *n* = 74; (L) first words, *n* = 63; (M) toilet trained stool, *n* = 44. ∗Percentiles of mortality are based on data derived from the Human Mortality database as reported by Beltrán-Sánchez et al^15^ and Verguet et al.^16^ Percentiles of developmental milestones are based on normative data of children without developmental delay.^17^^,^^18^ ID, intellectual disability; IQ, intellectual quotient; SDS, standard deviation score.

Eighty-seven adult-aged patients with pathogenic variants in *ARID1B* were included. For all patients, an online clinical survey was completed by the clinician. In 53 cases (61%) parents also completed an online questionnaire about their child. Forty-five patients have previously been published, and in 38 of them (84%) clinical data were updated (Supplemental Table 1). Facial photographs were available for 55 patients, and parents or caretakers provided consent for publication for 38 patients (Figure 1A and Supplemental Figure 1).

### Genotype

Figure 1B and Supplemental Table 1 give an overview of the *ARID1B* variants in our cohort. In 6 cases, the variant was passed on to 1 or more children (currently < 18 years of age, therefore their data was not included in this manuscript). Two of these patients had a mosaic variant. In 62 patients the pathogenic variant was *de novo*. In the remaining 19 patients, inheritance could not be determined or only 1 parent was available for testing. One inherited variant (ie., NM_020732.3:c. c.63_73del p.(Glu22Glnfs∗206)) was identified through exome sequencing and was initially classified as a variant of uncertain significance due to its location early in the transcript. Additional DNA methylation analysis on DNA derived from blood showed a BAFopathy episignature (Supplemental Figure 2), after which this variant was reclassified as pathogenic. All patients have variants predicted to lead to haploinsufficiency. A prior suspicion of CSS was present in 22.8% (18/79) of the cases. Figure 1B shows the distribution of pathogenic variants and degree of ID. Aside from the pathogenic variants in *ARID1B,* no other pathogenic deletions, duplications, or single nucleotide variants were identified in our patient cohort.

### Phenotype

An overview of patients’ characteristics is given in Table 1 and Supplemental Table 2. Ages ranged from 18 to 69 years with a median of 23.3 years. One patient died at the age of 24 years due to renal abscesses, and 1 patient died at the age of 47.2 years by asphyxiation due to choking on food (see also Figure 1C).

**Table 1:** Clinical characteristics of *ARID1B* patients

Patient Groups:	18+ (LoF)				LoF Exon 1		LoF > Exon 1		Mosaic	
*Clinical Features ^+^*	*n* = 85	%	*P Value*^a^	*Test*^a^	*n* = 16	%	*n* = 69	%	*n* = 2	%
Age (nr, min-max)	85	(18-69)	0.64	T	16	(18,5-48,5)	69	(18-69)	2	(35-37)
Sex (female)	85	61%	0.78	Chi	16	56%	69	62%	2	50%
Died	85	2%	1.00	F	16	0%	69	3%	2	0%
*Growth parameters & development*										
Gestational age, weeks (mean)	73	224	0.19	T	11	275	62	213	1	-
Birthweight (<-2 SDS)	53	11%	0.58	F	7	0%	46	13%	0	-
Height at birth (<-2 SDS)	16	31%	1.00	F	2	0%	14	36%	0	-
OFC at birth (<-2 SDS)	18	6%	1.00	F	3	0%	15	7%	0	-
Age last measurements, years (nr, min-max)	83	(4,2-69)	0.59	T	16	(11,8-40)	67	(4,2-69)	2	(35-37)
Weight (<-2 SDS)	55	0%	-	-	6	0%	49	0%	0	-
BMI >25 kg/m² or overweight	70	56%	1.00	F	10	60%	60	52%	1	100%
Length (<-2 SDS)	69	54%	0.49	F	9	67%	60	52%	2	50%
OFC (<-2 SDS)	55	5%	1.00	F	9	0%	46	7%	1	0%
Motor delay	78	92%	0.01	F	14	71%	64	97%	2	0%
Motor skills gross, delayed	78	76%	0.31	F	14	64%	64	78%	2	0%
Motor skills fine, delayed	78	65%	0.07	F	14	43%	64	70%	2	0%
Speech, delayed	81	98%	0.35	F	15	93%	66	98%	2	0%
Sleeping problems	59	36%	0.24	F	8	13%	51	39%	1	0%
Obstructive sleep apnea	85	1%	1.00	F	16	0%	69	1%	2	0%
Laryngomalacia	53	9%	1.00	F	10	10%	43	9%	2	0%
Feeding difficulties	81	65%	0.03	Chi	15	40%	66	71%	2	0%
Duration of feeding problems	42		0.17	F	5		37		0	
Brief		40%				60%		38%		-
Several years		38%				0%		43%		-
Ongoing		21%				40%		19%		-
Recurrent infections	71	39%	0.75	F	12	33%	59	41%	1	0%
Upper airway tract	71	4%	1.00	F	12	0%	59	5%	1	0%
Lower airway tract	71	3%	1.00	F	12	0%	59	3%	1	0%
ENT infections	71	17%	0.68	F	12	8%	59	19%	1	0%
Otitis media	71	14%	0.67	F	12	17%	59	14%	1	0%
Urinary tract	71	8%	1.00	F	12	8%	59	8%	1	0%
*Neurological features*										
IQ (nr, min-max)	32	(20-80)	0.48	T	4	(54-65)	28	(20-80)	0	-
Intellectual disability	85	93%	0.08	F	16	81%	69	96%	2	0%
Borderline	0	7%				19%		4%		50%
Mild	0	28%				38%		26%		0%
Mild-moderate	-	-	-	-	-	-	-	-	-	-
Moderate	0	42%				31%		45%		0%
Moderate-severe	-	-	-	-	-	-	-	-	-	-
Severe	0	20%				13%		22%		0%
Profound		0%				0%		3%		0%
Hypotonia	74	77%	0.13	F	12	58%	62	81%	2	0%
Seizures	81	47%	0.67	F	14	36%	67	49%	2	0%
No seizures, but abnormal EEG		7%				7%		7%		0%
Still experiencing seizures	20	15%	0.15	F	1	100%	19	11%	0	-
Loss of skill	64	25%	0.45	F	10	0%	54	30%	2	0%
Motoric	0	11%				0%		13%		0%
Speech	0	8%				0%		9%		0%
Unspecified	0	8%				0%		9%		0%
Agenesis of the corpus callosum	58	47%	0.94	F	8	50%	50	46%	2	0%
Partial/hypoplasia		31%				38%		30%		0%
Brain abnormality	67	55%	1.00	F	10	60%	57	54%	2	50%
MRI performed	78	85%	0.05	Chi	15	67%	63	89%	1	100%
*Vision and hearing impairments*										
Vision impaired	82	83%	1.00	F	15	87%	67	82%	2	0%
Myopia	60	78%	1.00	F	12	83%	48	77%	0	-
Hypermetropia	53	26%	0.09	F	8	0%	45	31%	0	-
Cataract	43	7%	1.00	F	7	0%	36	8%	1	0%
Hearing loss	79	28%	1.00	F	15	27%	64	28%	2	0%
Hearing loss, conductive	79	14%	1.00	F	15	13%	64	14%	2	0%
Hearing loss, perceptive	79	8%	0.59	F	15	0%	64	9%	2	0%
Eartubes	56	41%	1.00	F	11	36%	45	42%	2	0%
Hearing aid	17	35%	1.00	F	3	33%	14	36%	0	-
*Musculoskeletal anomalies*										
Orthopedic anomalies (scoliosis+patella+pes pedes)	85	61%	0.16	Chi	16	44%	69	65%	2	50%
Scoliosis	82	30%	1.00	F	15	27%	67	31%	2	50%
Degree scoliosis	3	(37-75)	-	-	0	-	3	(37-75)	0	-
Operation scoliosis needed	22	32%	0.52	F	3	0%	19	37%	1	0%
Pes planus	46	67%	0.65	F	6	83%	40	65%	2	50%
Patella luxation	45	27%	0.84	F	8	13%	37	30%	1	0%
Recurrent	12	67%			1	100%	11	64%		
Pectus, excavatum	80	3%	1.00	F	15	0%	65	3%	1	0%
Primary dentition, delayed	47	23%	0.66	F	9	11%	38	26%	2	0%
Permanent dentition, delayed	47	40%	0.28	F	9	22%	38	45%	2	0%
Widely spaced teeth	47	15%	1.00	F	9	11%	38	16%	2	0%
Abnormal dentition	36	69%	1.00	F	4	75%	32	69%	2	50%
Dental surgeon operation/treated by a dental surgeon	34	50%	0.38	F	5	20%	29	55%	0	-
Joint laxity	46	50%	0.24	F	8	25%	38	55%	2	0%
Early arthritis	33	6%	1.00	F	7	0%	26	8%	1	0%
Clinodactyly	65	12%	0.63	F	12	17%	53	11%	2	0%
Brachydactyly fifth finger	65	25%	1.00	F	12	25%	53	25%	2	0%
Small nails	66	38%	1.00	Chi	13	46%	53	36%	2	0%
Which nails, 5th finger, and/or toe	66	29%	0.17	Chi	13	46%	53	25%	2	0%
*Intestinal*										
Inguinal hernia	53	8%	0.54	F	9	11%	44	7%	1	0%
Intestinal problems	73	42%	0.54	F	13	31%	60	45%	1	0%
Constipation	73	27%	0.10	F	13	8%	60	32%	1	0%
Gastroesophageal reflux	73	11%	0,63	F	13	15%	60	10%	1	0%
Diarrhea	73	0%	-	-	13	0%	60	0%	1	0%
Pyloric Stenosis	73	0%	-	-	13	0%	60	0%	1	0%
Umbilical hernia	73	4%	0.08	F	13	15%	60	2%	1	0%
*Cardiac & urogenital anomalies*										
Cardiac anomalies	65	14%	1.00	F	12	8%	53	15%	1	0%
ASD	65	6%	1.00	F	12	0%	53	8%	1	0%
VSD	65	0%	-	-	12	0%	53	0%	1	0%
Aortic valve abnormality	65	3%	0.34	F	12	8%	53	2%	1	0%
Mitralis insufficiency	65	2%	1.00	F	12	0%	53	2%	1	0%
Renal anomalies	42	43%	0.01	F	6	0%	36	50%	0	-
Hydronephrotic kidney	42	10%	1.00	F	6	0%	36	11%	0	-
Nephrolithiasis	42	21%	0.31	F	6	0%	36	25%	0	-
Renal sonography, abnormal	43	42%			6	0%	37	49%	0	-
Age identification of first renal stone (nr, min-max)	5	(7-59)	-	-	0	-	5	(7-59)	0	-
Cryptorchidism	30	60%	0.66	F	6	50%	24	63%	1	0%
Endocrinological abnormalities										
Diabetes mellitus	54	11%	1.00	F	8	13%	46	11%	0	-
Type 2 diabetes mellitus	54	11%	1.00	F	8	13%	46	11%	0	-
Hypothyroidism	54	15%	1.00	F	8	13%	46	15%	0	-
Growth hormone deficiency	54	2%	1.00	F	8	0%	46	2%	0	-
Other										
Anemia	54	6%	1.00	F	8	0%	46	7%	0	-
Elevated cholesterol	54	7%	1.00	F	8	0%	46	9%	0	-
Hypertension	35	17%	0.56	F	5	0%	30	20%	0	-
Behavioral abnormalities	80	85%	0.40	F	13	77%	67	87%	2	50%
Hyperactivity	75	7%	0.59	F	13	0%	62	8%	2	0%
High pain threshold	53	64%	0.26	F	9	44%	44	68%	1	0%
Psychiatric disorders										
ADHD	80	9%	1.00	F	13	8%	67	9%	2	50%
Autistic traits	80	26%	0.50	F	13	15%	67	28%	2	0%
Autism	80	31%	0.75	F	13	23%	67	33%	2	0%
Age autism diagnosis (nr, min-max)	21	(0-25)	0.89	MW	3	(3-12)	18	(0-25)	0	-
Auto-mutilation	80	19%	0.11	F	13	0%	67	22%	2	0%
Malignancies	73	1%	1.00	F	12	0%	61	2%	2	0%
Lifestyle										
Daycare	49	65%	0.15	F	7	57%	42	67%	0	-
Regular	0	22%				43%		19%		
Special	0	43%				14%		48%		
Primary education	59	100%	0.15	F	8	100%	51	100%	0	-
Regular		2%				13%		0%		
Special		69%				88%		67%		
Secondary education	45	73%	0.38	F	7	86%	38	71%	0	-
Regular	0	67%				14%		5%		
Special	0	0%				71%		66%		
Living situation	57		0.22	F	8	0%	49	0%	0	-
At home/with parents		67%				75%		65%	0	-
Independently guided/assisted living		9%				13%		8%	0	-
Residential group (>residents/caretaker)		19%				0%		22%	0	-
Residential group (1 on 1 guidance)		5%				13%		4%	0	-
Medication	65	74%	0.69	F	9	67%	56	75%	2	100%
Anti-epileptics	65	23%	1.00	F	9	22%	56	23%	2	0%
Anti-depressants	65	12%	0.31	F	9	22%	56	11%	2	50%
Anti-psychotics	65	12%	0.59	F	9	0%	56	14%	2	0%
Diuretics/Anti-hypertensives	65	12%	1.00	F	9	11%	56	13%	2	50%
Amphetamines	65	5%	1.00	F	9	0%	56	5%	2	0%
Anti-diabetics	65	8%	1.00	F	9	0%	56	9%	2	0%
Hypo-/hyperthyroidism medication	65	8%	0.14	F	9	22%	56	5%	2	50%
Laxatives	65	15%	0.33	F	9	0%	56	18%	2	0%
PPI	65	11%	0.58	F	9	0%	56	13%	2	0%
Other	65	43%	0.07	F	9	11%	56	48%	2	50%

A, ANOVA; ADHD, attention deficit hyperactivity disorder; ASD, Atrial Septal Defect; BMI, body mass index; Chi, Chi-square; EEG, electro encephalography; ENT, ear nose throat; F, Fisher's exact; KW, Kruskal-Wallis; LoF, loss of function variants; MW, Mann-Whitney U; OFC, occipital–frontal circumference; SDS, standard deviation score; T, T-test; VSD, ventricular septal defect.

^+^ the total number of a feature can differ from the sum of subcategories because in some cases it was possible to answer with more than 1 option or to report the existence of a feature without specifying.

a Groups compared are patients with a pathogenic variant in exon 1 versus patients with an exon 2-20 variant or a deletion in *ARID1B*.

Frequencies reported henceforth concern the 85 non-mosaic patients.

### Congenital anomalies

Frequently reported congenital anomalies are agenesis of the corpus callosum (27/58, 47%), cardiac anomalies (9/65, 14%), renal abnormalities (18/42, 43%), and cryptorchidism (18/30, 60%).

### Growth

Birthweight below 2 SDS was observed in 11% (6/53) of patients (Figure 1D), and feeding difficulties were reported in 65% (53/81). For the majority, feeding issues started at birth (74%, 34/46) and were transient in 79% of cases (brief: 40%, 17/42; several years: 38%, 16/42), with 21% (9/42) experiencing ongoing difficulties.

Histograms of the SDS of height, weight, and occipital-frontal circumference (OFC) are shown in Figure 1E-G. The majority of patients have a height below 0 SDS (97%, 67/69); 56% (39/70) have a body mass index above 25 kg/m² or are reported to be overweight; OFC is distributed normally around 0 SDS.

### Development

Ninety-three percent of patients have ID (Table 1, Figure 1H-I), with total IQ scores (*n* = 32) ranging from 20-80 (Figure 1I). Eight patients had borderline or normal intelligence (i.e. an estimated normal intelligence or an IQ score of 80 or higher). IQ values were available for only 3 patients with an estimated borderline or normal IQ score. One patient had a total IQ of 80 measured at the age of 14 years, another patient had a verbal IQ of 92 and a performance IQ of 70 at the age of 18 years, and the last patient had a verbal IQ of 83 and a performance IQ of 70 at an unknown age. Of the 8 patients with a borderline or normal IQ, 5 had behavioral anomalies: attention deficit hyperactivity disorder in 4 patients and autistic features in 2 patients. Self-mutilation was not reported in this group.

Hypotonia was observed in 77% (57/74). Figures 1J-L show developmental milestones. Motor and speech are delayed in most patients. While almost all patients eventually walk, approximately one-third of patients do not develop speech. Seventy-five percent of patients are toilet trained (Figure 1M, Supplemental Figure 3A). Fifty-five patients can read, and 33 patients can write. The age of puberty onset (*n* = 39) varied between 9 and 21 years (Supplemental Figure 3B).

**Figure 2:**
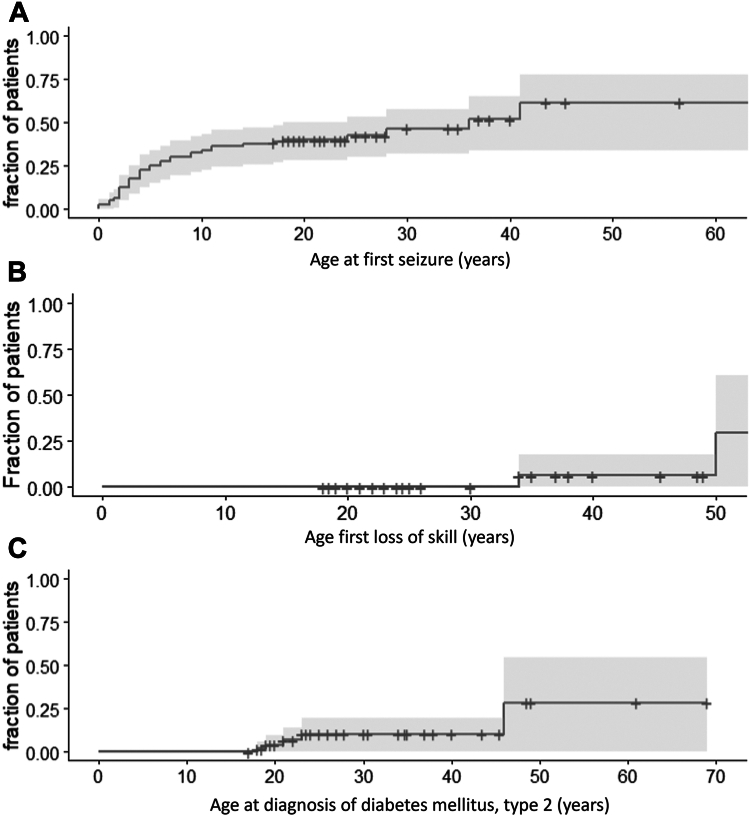

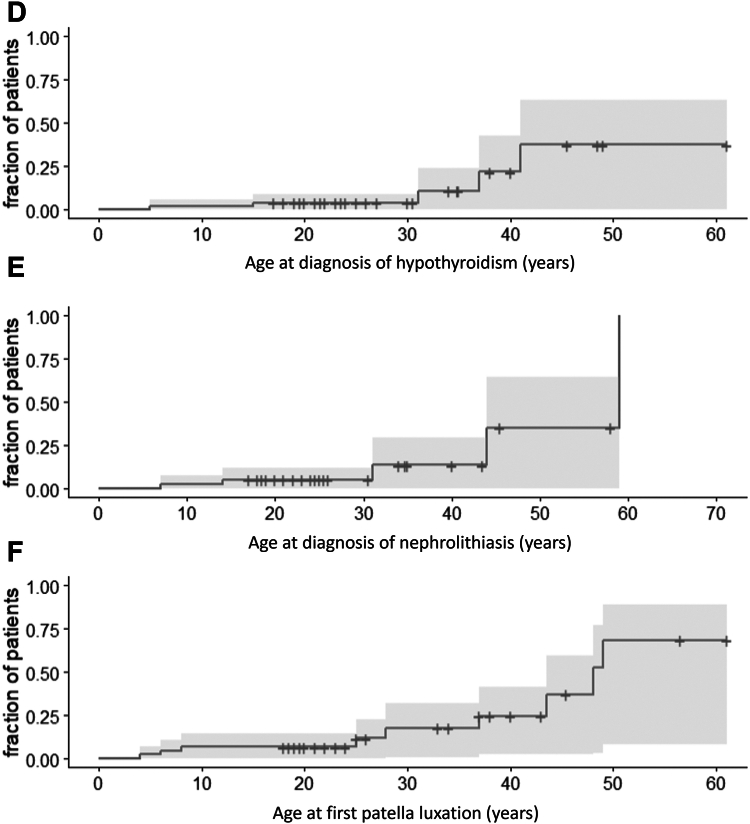
**Survival analysis of features developing later in life in adult*****ARID1B*****patients.** Kaplan-Meier plot for the age of (A) onset first seizure, *n* = 80; (B) loss of skill, *n* = 54; (C) diabetes mellitus, type 2, *n* = 53; (D) hypothyroidism, *n* = 51; (E) nephrolithiasis, *n* = 38; (F) first patella luxation, *n* = 43.

Seizures occurred in 47% (38/81) of patients, with an additional 7% (6/81) with an abnormal electroencephalogram (EEG). The age of onset (*n* = 80) varied between 0 and 41 years (Figure 2A). Loss of skills was noted in 25% (16/64) of patients. No specific triggering event was reported. The age of onset was documented in 4 cases. This age ranged from 31 to 54 years (Figure 2B). Loss of motor skills, particularly in walking ability (with increased tripping and decreased balance) was the most common (7/16), followed by loss of speech (5/16). Some patients required the use of a wheelchair (5/62).

### Vision and hearing

Most patients had impaired vision 83% (68/82), usually due to myopia (78%, 47/60). Myopia severity (*n* = 27) ranged from -1 to -25 (Supplemental Figure 3C), with 14 patients having a severity below -10. Hearing loss was present in 28% (22/79) of patients; with 6 patients needing a hearing aid. Both conductive and perceptive hearing impairment are reported, with conductive hearing impairment being slightly more prevalent (Supplemental Table 2).

### Other features

Diabetes mellitus type 2 was reported in 11% (6/54) of patients (Figure 2C), hypothyroidism in 15% (8/54) of patients (Figure 2D), and nephrolithiasis in 21% (9/42) (Figure 2E). Recurrent infections were present in 39% (28/71) of patients. Anemia was identified in 6% (3/54), elevated cholesterol in 7% (4/54), and hypertension in 17% (6/35) of patients. Sleeping problems were reported in 36% (21/59).

Behavioral anomalies were reported by clinicians in 85% (68/80). Seventy-five percent (36/48) of parents report the behavior of their child as being problematic (“sometimes” 25/48 [52%] or “often” 11/48 [23%]). Auto-mutilation was reported in 19% (15/80) of patients.

Abnormal dentition was noted in 69% (25/36) of patients, and 50% (17/34) received treatment from a dental surgeon. Although the specific reasons for dental treatment were not explicitly asked, it was reported that several patients required the extraction of multiple teeth.

Approximately one-third (30%, 25/82) of patients developed scoliosis, with 32% of those (7/22) requiring surgery. Pes planovalgus was reported for 67% (31/46), and 60% (25/42) of patients used support insoles. Talocalaneonavicular dislocation was reported in the surveys’ open fields. Patella luxation (Figure 2F) was reported in 12/45 patients, and in 8 cases these were recurrent. Patella luxation occurred at ages between 4 and 49 years with a median of 27.9 years. At least 3 patients were reported to have undergone surgery for recurrent patella luxation, as noted in the free text, although no specific question regarding this surgery was included.

One patient in our cohort was reported to have a lung tumor, but based on radiological examination and consultation with a pulmonologist, it was determined to be likely benign. Therefore, a biopsy was deemed to be overly burdensome in this patient with severe ID.

### Functioning

Table 2 and Supplemental Table 3 show to what extent the included adult patients with *ARID1B*-related disorder were able to perform activities of daily living based on our parental questionnaire. Many (45/51, 88%) patients were able to eat and drink independently, 73% (37/51) were able to dress without help, 30% (15/50) independently shop for groceries and 8% (4/49) could prepare dinner independently. Sixty-five percent of the patients (30/46) could stay home alone for 30 minutes, and 16% (7/45) could travel alone by public transport. Most patients lived at home with parents (38/57, 67%), 9% of patients (5/57) lived in assisted living, and the remaining patients lived in groups or had 1 on 1 guidance.

**Table 2:** Activities of daily living: parent-reported outcomes for their adult children

	18+ (LoF)				LoF patients exon 1		LoF patients >exon 1	
	*n* = 85	%	*P* Value^a^	Test	*n* = 16	%	*n* = 69	%
Can your child make his/her own bed?	49		0.48	F	7		42	
With help		53%				57%		52%
Independently		31%				43%		29%
Can your child clean up, and do light housework?	51		0.73	F	7		44	
With help		59%				71%		57%
Independently		27%				29%		27%
Can your child do the groceries?	50		0.06	F	7		43	
With help		34%				43%		33%
Independently		30%				57%		26%
Can your child replace a lamp, or tighten a screw?	50		0.00	F	7		43	
With help		32%				71%		26%
Independently		14%				29%		12%
Can your child do the laundry?	49		0.03	F	7		42	
With help		47%				57%		45%
Independently		16%				43%		12%
Can your child take a bath or shower?	51		0.05	F	7		44	
With help		51%				14%		57%
Independently		41%				86%		34%
Can your child brush his/her teeth and comb his/her hair?	51		0.19	F	7		44	
With help		47%				29%		50%
Independently		37%				71%		32%
Can your child dress and undress him/herself?	51		0.26	F	7		44	
With help		22%				0%		25%
Independently		73%				100%		68%
Can your child go to the toilet?	51		0.74	F	7		44	
With help		16%				0%		18%
Independently		78%				100%		75%
Can your child make sandwiches?	51		0.16	F	7		44	
With help		29%				14%		32%
Independently		51%				86%		45%
Can your child fry an egg, make pancakes, or heat food in the microwave	50		0.25	F	7		43	
With help		48%				57%		47%
Independently		16%				29%		14%
Can your child prepare dinner?	49		0.02	F	7		42	
With help		37%				57%		33%
Independently		8%				29%		5%
Can your child set and clear the table?	50		0.41	F	7		43	
With help		30%				14%		33%
Independently		58%				86%		53%
Can your child drink from a cup?	51		1.00	F	7		44	
With help		0%				0%		0%
Independently		96%				100%		95%
Can your child eat from a plate?	51		1.00	F	7		44	
With help		8%				0%		9%
Independently		90%				100%		89%
Can your child do the dishes or load the dishwasher?	47		0.64	F	7		40	
With help		30%				29%		30%
Independently		55%				71%		53%
Can your child handle money, and pay in the store?	46		0.21	F	7		39	
With help		35%				43%		33%
Independently		13%				29%		10%
Can your child stay home alone for 30 minutes?	46		0.39	F	7		39	
Yes		65%				86%		62%
Can your child travel alone by public transport?	45		0.30	F	7		38	
Yes		16%				29%		13%

F, Fisher's exact; LoF: loss of Function variants.

a Groups compared are patients with a pathogenic variant in exon 1 versus patients with an exon 2-20 variant or a deletion in *ARID1B*.

### Medication use

Medication is utilized by 74% (48/65) of patients in the cohort. Among the reported medications, anti-epileptic drugs (23%, 15/65) are the most frequently used, followed by laxatives (15%, 10/65), anti-depressants (12%, 8/65), and anti-psychotics (12%, 8/65) (Table 1). A combination of antidepressant or antipsychotic medication is used by 20% (13/65) of patients. Other medications include antihypertensive drugs (12%, 8/65), anti-diabetics (8%, 5/65), and medication for hypo/hyperthyroidism (8%, 5/65). Response to medication was assessed only for seizure medication. Frequently prescribed anti-convulsive medications were valproic acid (*n* = 5), lamotrigine (*n* = 3), carbamazepine (*n* = 3), and, levetiracetam (*n* = 3). Ninety percent of patients (18/20) responded well to anti-convulsive therapy.

### Variants in exon 1 lead to a milder phenotype than deletions or variants in exon 2-20

All pathogenic *ARID1B* variants inherited from non-mosaic parents are located in exon 1 (Figure 1A). Among patients with exon 1 variants, 19% (3/16) exhibit borderline to no ID; in comparison, only 4% (3/69) of individuals in the exon 2-20 group are described as having borderline to no ID (*P* = .07). In addition, exon 1 patients tend to have less fine motor delay (*P* = .01), fewer feeding difficulties (*P* = .03) and no reported renal anomalies (*P* = .01) (Table 1, Supplemental Table S2, Supplemental Figure 4). On all activities of daily living mentioned in Table 2, a higher proportion of patients with the exon 1 variant score as ‘independent’, indicating that they have a higher level of self-sustainability. For example, 86% of patients with exon 1 variants can take a bath or shower independently compared to 34% in the exon 2-20 and whole gene deletion group (*P* = .05).

### Facial features

Using the most recent facial photographs, patients with *ARID1B*-related disorders (*n* = 48) were distinguishable from age and sex-matched NDD controls (analysis 1: *P* < .01) (Supplemental Table 4, Supplemental Figure 5). This distinction held true when stratified by age groups (0-4, 5-10, 11-17, 18-25, and 25+ years) (analysis 2: *P* < .01). Notably, the age group 11-17 years displayed the lowest Brier score and the highest area under the curve (AUC). Using 14 facial photos of patients aged below 11 years and the same 14 patients aged above 25 years, the photos of these patients aged below 11 years were more significantly different from controls (analysis 3a: *P* < .01) compared to photos of these patients aged above 25 years (analysis 3b: *P* = .05). Facial photos of 8 patients with variants in exon 1 were not distinguishable from NDD controls (analysis 4a: *P* = .65), while the 43 photos of patients with variants outside exon 1 or whole gene deletions differed from NDD controls (analysis 4b: *P* < .01). Additionally, when comparing the 7 photos (one photo could not be age-matched) of patients with exon 1 variants to those with variants in other exons, no significant difference was observed (analysis 4c: *P* = .84).

Facial photos of patient 066, who had a mosaic *ARID1B* pathogenic variant, were analyzed at different ages (0.6, 4, 10, 18, and 40 years) and were found to cluster with photos of patients with non-mosaic pathogenic variants (Supplemental Table 5). When comparing each photo to the photos of patients and controls in the corresponding age group, those taken at ages 11 and 17 years exhibited the most consistent clustering patterns.

## Discussion

We report the first adult-aged cohort of 87 patients with pathogenic *ARID1B* variants. We confirmed our previous hypothesis^11^ that patients with variants predicted to lead to haploinsufficiency in exon 1 tend to have a milder ID phenotype. In addition, we determined that (adult-aged) patients with *ARID1B*-related disorder have a risk of recurrent patella luxation, loss of skills, and auto-mutilation. Additionally, we confirmed our previous findings that patients with *ARID1B-*related disorder have a risk of seizures, myopia, nephrolithiasis, hypothyroidism, diabetes mellitus type 2, and scoliosis.

### Genotype

All patients in our cohort have variants predicted to lead to haploinsufficiency. Some pathogenic missense variants have been reported in literature,^8^^,^^21^, ^22^, ^23^ but they are much less common than predicted loss-of-function variants. Further research is necessary to study these missense variants and determine if patients carrying such variants differ from those with predicted loss-of-function variants, which have been shown to lead to nonsense-mediated decay on several occasions.^24^, ^25^, ^26^ Further studies are needed to confirm that nonsense-mediated decay is happening with most predicted loss-of-function variants versus the formation of truncated proteins.

Most of the variants occurred *de novo*, but several variants were inherited. All variants inherited from nonmosaic parents were located in exon 1. In retrospect, these parents have several features fitting with *ARID1B*-related disorder, indicating a full penetrance of these variants.^27^

### Genotype-phenotype

As shown in Figure 1B, patients with pathogenic variants in exon 1 of *ARID1B* tend to have milder ID. It is hypothesized that variants at the start of exon 1 may not be pathogenic.^5^^,^^11^ Based on our current data, we conclude that predicted loss-of-function variants in exon 1 are pathogenic, but tend to lead to a milder phenotype compared to variants located further on the transcript.

This phenomenon might be explained by a partial rescue because of an alternative start site.^28^ For example, in the GTEx portal^29^ there are several transcripts starting later than the exon 1 start site of NM_020732.3, and there are also several transcripts starting before exon 1 start site of NM_020732.3.

### Phenotype

This study provides the first comprehensive assessment of the self-sustainability of adult-aged patients with pathogenic *ARID1B* variants. Parents of newly diagnosed individuals often inquire about the extent to which this patient group is toilet trained, able to read and/or write, has received education, and can perform activities of daily living, and those questions can now be answered.

Our study confirms the wide spectrum of individuals with *ARID1B*-related disorders. This spectrum includes individuals without ID who live independently, typically associated with very early exon 1 variants or mosaic variants. On the other end of the spectrum are individuals with severe ID requiring 24-hour care. Between these extremes, we found patients with varying levels of independence (Table 2).

We also confirmed that height in *ARID1B* patients is lower than that of the general population with an average SDS of -2.0. We used published growth charts to impute missing SDS. These growth charts are based on a predominantly White population. It could, therefore, be possible that in the 7 cases with a mixed or non-White ancestry SDS for height was overestimated. However, based on these growth charts, 3 of 7 patients had a height below -2 SDS, which is similar to the distribution of the group as a whole. Myopia (78%) and hypermetropia (26%) are more prevalent in our population, compared with the general population where myopia is present in 4.9% to 18.2% and hypermetropia in 2.2% to 14.3%.^30^ Age of puberty onset is at an average of 14.7 years, with several outliers starting at the age of 17 to 21 years. These numbers are based on both clinician and parent reports.

New features identified in this cohort are recurrent patella dislocation and loss of skill. Recurrent patella luxation can have several causes, including weakness of the thigh muscles, or excess pronation of the feet. Both factors can play a role in our population as many patients (77%) experience hypotonia, 67% have pes planovalgus and in 1 patient, a talocalaneonavicular dislocation has been reported. The loss of skills observed in 25% of patients is important to consider when caring for a patient with a pathogenic *ARID1B* variant. This number may be biased, as loss of skills may be the reason to refer an adult to a genetic center for diagnostic evaluation. Further investigation is required to assess whether timely interventions can, for example, improve functioning or aid in restoring motor function. In instances where the onset of loss of skills can be linked to a specific event, eye movement desensitization and reprocessing therapy may offer valuable support.

Interestingly, although feeding problems are frequent in childhood, we observe that over half of our adult cohort is overweight (body mass index > 25 kg/m², or as reported by a parent). People with intellectual disabilities are generally less physically active,^31^ and research has demonstrated the benefits of exercise in improving cardiorespiratory and muscular fitness.^32^ Given the correlation of obesity and type 2 diabetes (4/5 individuals with this disease had obesity in our cohort), on indication, diabetes should be tested. This also goes for other features that develop with age (Supplemental Tables 6-7), and therefore, we have formulated screening recommendations (Table 3). Furthermore, it is noteworthy that aside from laxatives, anti-epileptic drugs, anti-depressants, and anti-psychotics are the most prescribed medications in our patient group, reflecting the impact of behavior on daily living and patient management.

**Table 3:** Recommendations surveillance guidelines *ARID1B* patients

Evaluation (Inquire/Perform physical examination)	After diagnosis	Age category			(para) medic^a^
		*Children (0-18 years)*	*Adults (18+)*	Frequency	
Congenital abnormalities (heart, kidneys and cryptorchidism)	Yes	Upon indication		Upon indication	Treating physician
Feeding difficulties	Yes	Yes		At every visit	Pediatrician
Constipation	Yes	Yes	Yes	At every visit	Treating physician
Growth and weight	Yes	Yes	Yes	At every visit, to avoid excessive weight gain	Pediatrician, dietitian
Seizures	Yes	Yes	Yes	At every visit	Treating physician
Endocrine/hormonal	Upon indication	Upon indication	At every visit^b^	Upon indication or every 3 years if there are risk factors (i.e. overweight)	Treating physician
Vision	Yes	Yes	Yes	Every 2 years, the frequency can be adjusted if the patient is able to report on eyesight	Ophthalmologist
Hearing	Yes	Yes	Upon indication	At every visit ask for signs of hearing loss and refer to an audiologist if suspected	Treating physician
Nephrolithiasis	Yes	Yes	Upon indication	At every visit	Treating physician/urologist
Scoliosis	Yes	Yes	Upon indication	Periodically, until length growth is complete	Orthopedic surgeon/ physiatrist/ pediatrician
Patella luxation	Yes	Upon indication	Upon indication	Upon indication	Orthopedic surgeon/ physiatrist/ pediatrician
Pedes (plano)valgi	Yes	Yes	Upon indication	Periodically	Orthopedic surgeon/ physiatrist/ pediatrician
Dentition/dental health	Yes	Yes	Yes	Twice a year	Dentist
Motor development and/or loss of skill	Yes	Yes	Upon indication	At every visit	Pediatrician/ physiatrist/ physical therapist/ treating physician
Cognitive development	If aged <16	Yes		At every visit	Pediatrician/ psychologist
Communication/language development and/or loss of skill	Yes	Yes	Upon indication	Screening at age 2 and 3 years, then on indication	Pediatrician/ physiatrist/ speech therapist/ treating physician
Behavioral and social development/impairments	Yes	Yes	Yes	At every visit	Treating physician
Sexual development	When applicable	Yes	Yes	When applicable	Treating physician
Transition to adult care	When applicable	When applicable		From the age of 16 years	Physiatrist/ pediatrician

a treating physician (eg, general practitioner, pediatrician).

b especially, glucose and thyroid lab.

We also noted some differences with current frequency estimates, compared to our previous work. For example, in one publication^5^ 75% of patients had developed speech by the age of 5 years. In our cohort, only 60% of patients have developed speech by the age of 5 years, although this estimate rises toward 75% at the age of 7 years (Figure 1L). This difference may be caused by random variation, but may also point to a more severely affected cohort because of either ascertainment bias (genetic testing is more often done in more severely affected adult-aged patients) or a reduced quality of care in this older cohort. Similarly, Figure 2A indicates that 60% will develop epilepsy whereas previously this estimate was 35%.^5^ This difference seems to be caused by a substantial proportion having their first seizure after the age of 20 years, which we previously missed because of reduced follow-up.

Somatic variants in *ARID1B* have been associated with several types of cancer.^33^ In the literature only occasional cases of *ARID1B* patients with cancer are reported.^5^^,^^34^ We did not identify an increased cancer risk in our cohort. Based on our cohort and literature, there is no indication that germline variants in *ARID1B* give an increased cancer risk at pediatric age. Although there is currently no evidence to suggest an elevated cancer risk in adult-aged patients, it is important to acknowledge that our study had a limited representation of patients over the age of 50 years, and further longitudinal research is needed to confirm this.

### Pediatric versus adult cohort

Compared to the previously published predominantly pediatric cohort^5^ (Supplemental Figure 6), our adult cohort exhibits a notable shift in phenotype (Supplemental Table 6-7). One example is the previously mentioned shift from feeding difficulties in children to overweight in adults. Similarly, recurrent infections are significantly less prevalent in our adult cohort (39% compared to 57%,^5^ *P* = .03). In children with *ARID1B*-related disorder, the primary emphasis often revolves around development and the acquisition of new skills. However, as these patients transition into adulthood, the focus shifts towards the preservation of current skills or the prevention of loss of skills and maintenance of muscular and cardiorespiratory fitness. This divergence in focus underscores the evolving needs and priorities of individuals with this condition as they grow older.

### Facial photograph analyses

Our study demonstrated that *ARID1B* patients are distinguishable from matched NDD controls based on facial features in infancy. However, in our earlier analysis, there was an indication these distinctive facial features may become less specific as individuals with *ARID1B*-related disorders age,^11^ whereas in the current analysis, all age groups clustered separately from matched NDD controls. When using fewer photographs, we did see less significant clustering results for photos of patients aged 25+ years (Supplemental Table 5), indicating the difference in results from our previous analysis may be explained by the then limited number of available photos.

Nonetheless, if facial analysis is implemented to assist with interpretation, we recommend using childhood photographs, especially since most available photos are still of patients aged below 18 years. This approach can provide a more reliable basis for accurate diagnosis and interpretation.

### Limitations

Our study has several potential limitations that should be considered. One is that multiple clinicians contributed to data entry, which may have introduced variability and dataset inconsistencies. There is also a risk of overestimating the prevalence of certain features due to the unknown status regarding specific features in some patients as, in our experience, a clinician is more likely to tick the box “unknown” for a feature than “absent.” In addition, there may be ascertainment bias in our study towards more severe cases as genetic diagnostics may be performed more often on more severely affected adult patients. We tried to limit this by including as many adult-aged patients as we could find.

### Conclusion

The *ARID1B* spectrum is broad, and as patients age, there is a significant shift in the medical aspects that need attention. Several features warrant extra attention in adult patients, and screening and treatment of these features may prevent progression. Therefore, we have updated our screening recommendations^5^^,^^35^ for all age groups to promote timely intervention in an attempt to potentially improve health outcomes (Table 3).

## Data Availability

De-identified patient data will be made available on request to the corresponding author.

## Conflict of Interest

Jill A. Rosenfeld: The Department of Molecular and Human Genetics at Baylor College of Medicine receives revenue from clinical genetic testing completed at Baylor Genetics Laboratories. Evan E. Eichler is a scientific advisory board (SAB) member of Variant Bio, Inc. All other authors declare no conflicts of interest.

## Supplementary Material

Supplementary Tables and Figurs The supplementary material consists of 7 tables, 6 figures and 1 supplementary excel file that support the results reported in this article. All tables, figures and the excel are mentioned in the text. This supplementary material includes, but is not limited to, variable labels of the online questionnaire questions, genetic variants, facial photographs, extensive overviews of the clinical characteristics of the patients reported in this paper, PhenoScore analyses, and a comparison of our patient group with a previously published cohort of *ARID1B* patients.
