# Supplementary material for: ARID1B-related disorder in 87 adults: Natural history and self-sustainability
Source: Genet Med Open. 2024 Jul 23;2:101873. doi: 10.1016/j.gimo.2024.101873 (PMC11613905; doi:10.1016/j.gimo.2024.101873)
Supplement: Supplementary Tables and Figurs — The supplementary material consists of 7 tables, 6 figures and 1 supplementary excel file that support the results reported in this article. All tables, figures and the excel are mentioned in the text. This supplementary material includes, but is not limited to, variable labels of the online questionnaire questions, genetic variants, facial photographs, extensive overviews of the clinical characteristics of the patients reported in this paper, PhenoScore analyses, and a comparison of our patient group with a previously published cohort of ARID1B patients. [file mmc1.pdf]

SUPPLEMENTARY MATERIAL

***ARID1B*-related disorder in 87 adults: natural history, self-sustainability and new features leading to updated screening recommendations**

P.J. van der Sluijs, M. Gösgens, A.J.M. Dingemans, P. Striano, A. Riva, C. Mignot, A. Faudet, G. Vasileiou, M. Walther, S.A. Schrier Vergano, M. Alders, F.S. Alkuraya, I. Alorainy, H.S. Alsaif, B. Anderlid, I. Bache, I. van Beek, M. Blanluet, B.W. van Bon, T. Brunet, H. Brunner, M.L. Carriero, P. Charles, N. Chatron, E. Coccia, C. Dubourg, R.K. Earl, E.E. Eichler, L. Faivre, N. Foulds, C. Graziano, A.M. Guerrot, M.O. Hashem, S. Heide, D. Heron, S.E. Hickey, S.M.J. Hopman, A.A. Kattentidt-Mouravieva, J. Kerkhof, J.S. Klein Wassink-Ruiter, E.C. Kurtz-Nelson, K. Kušíková, M. Kvarnung, F. Lecoquierre, G.S. Leszinski, L. Loberti, P.L. Magoulas, F. Mari, I. Maystadt, G. Merla, J.M. Milunsky, S. Moortgat, G. Nicolas, M. O' Leary, S. Odent, J.R. Ozmore, K. Parbhoo, R. Pfundt, M. Piccione, A.M. Pinto, B. Popp, A. Putoux, H.L. Rehm, A. Reis, A. Renieri, J.A. Rosenfeld, M. Rossi, E. Salzano, P. Saugier-Veber, M. Seri, G. Severi, F.M. Sonmez, G. Strobl-Wildemann, K.E. Stuurman, E. Uctepe, H. Van Esch, G. Vitetta, B.B.A. de Vries, D. Wahl, T. Wang, P. Zacher, K.R. Heitink, F.G. Ropers, D. Steenbeek, T. Rybak, G.W.E. Santen

Content

Supplementary Table 1: Genetic variants identified in *ARID1B* patients p. 3

Supplementary Figure 1: *ARID1B* patient photos p. 5

Supplementary Figure 2: DNA methylation results case 677 p. 7

Supplementary Table 2: Clinical characteristics of ARID1B patients p. 8

Supplementary Figure 3: Cumulative distribution of toilet training*, histogram of age onset puberty and histogram of myopia severity p. 10

Supplementary Table 3: Activities of daily living: Parent reported outcomes for their adult children p. 12

Supplementary Figure 4: Kaplan-Meiers comparing patients with variant in exon 1 with variants > exon 1 p. 13

Supplementary Table 4: PhenoScore analyses – all patients p. 17

Supplementary Figure 5: Facial heatmaps of PhenoScore analyses using photos of all patients p. 18

Supplementary Table 5: PhenoScore analyses – mosaic case p. 21

Supplementary Figure 6: A) Age distribution in current *ARID1B* cohort B) Age distribution within published *ARID1B* cohort[1] p. 22

Supplementary Table 6: Clinical characteristics of *ARID1B* patients compared with cohort from van der Sluijs *et al.* 2018[1] p. 24

Supplementary Table 7: Clinical characteristics of *ARID1B* patients and cohort from van der Sluijs *et al.* 2018[1] with a statistical comparison p. 25

*Percentiles of control population are based normative data of children without developmental delay[2,3]

Supplementary Table 1: Genetic variants identified in *ARID1B* patients

Patient ID	Genomic location (GRCh37) NC_000006.11:	cDNA (NM_020732.3)	Protein change	Inheritance	Technique	Parent/via child identified	Mosaic	Published	Published phenotype data	Data updated	Parental questionnaire	Facial photo available	Consent publication	Published patient number
255	g.(?_151019422)_(159187660_?)del	c.-6079642_*1658635del	p.0	*de novo*	Array			Yes	Yes	Yes	Yes	Yes	Yes	Patient 7[2]
245	g.(152497968_152788282)_(157497968_157996910)del	c.-4601096_*467885del	p.0	*de novo*	Array			Yes	Yes	Yes		Yes	No	patient 6 (2012)[2], patient 17 (2022)[4]
576	g.(?_156657842)_(157966721_?)del	c.-441222_*437696del	p.0	*de novo*	Array			Yes	Aggregated	Yes	Yes	Yes	No	patient 137[1]
341	g.(?_157079676)_(157806675_?)del	c.-19388_*277650del	p.0	*de novo*	Array			Yes	Summier	Yes	Yes	Yes	Yes	subject 5[6]
677	g.157099126_157099136del	c.63_73del	p.(Glu22Glnfs*206)	not in mother	Exome (variant in child)	Yes		No			Yes	Yes	Yes	
345	g.157099913C>T	c.850C>T	p.(Gln284*)	*de novo*	Whole exome sequencing			Yes	Aggregated	Yes	Yes	Yes	Yes	patient 140[1]
187	g.157100092_157100119del	c.1029_1056del	p.(Ala349Metfs*11)	unknown	Exome (variant in child)	Yes		Yes	Summier	Supplemented		No		Case 5[7]
594	g.157100092_157100128del	c.1029_1065del	p.(Ala346Metfs*11)	unknown	Exome (variant in child)	Yes		No			Yes	Yes	No	
238	g.157100107_157100134del	c.1044_1071del	p.(Ala349Metfs*11)	unknown	Exome (variant in child)	Yes		Yes	Summier	Supplemented		Yes	No	Case 6[7]
652	g.157100107_157100134dup	c.1044_1071dup	p.(Tyr358Serfs*186)	*de novo*	Exome (variant in child)	Yes	Yes	No				No		
430	g.157100177dup	c.1114dup	p.(Arg372Profs*163)	*de novo*	Targeted diagnostics			Yes	Yes	No		No		patient 9[8]
486	g.157100209dup	c.1146dup	p.(Gly383Argfs*152)	*de novo*	Whole exome sequencing			No			Yes	Yes	No	
005	g.157100265del	c.1202del	p.(Gly401Alafs*29)	*de novo*	Whole exome sequencing			Yes	Aggregated	Yes	Yes	Yes	Yes	patient 133[1]
317	g.157100285dup	c.1222dup	p.(Gln408Profs*127)	*de novo*	Targeted diagnostics			Yes	Yes	No		Yes, published (supplementary)		patient 24 (2013)[5], patient 7 (2018)[1]
329	g.157100298dup	c.1235dup	p.(Ser413Valfs*122)	*de novo*	Targeted diagnostics			Yes	Yes	Yes	Yes	Yes	Yes	patient 55 (2013)[5], patient 39 (2018)[1]
316	g.157100322dup	c.1259dup	p.(Asn420Lysfs*115)	*de novo*	Targeted diagnostics			Yes	Yes	No		No		patient 19 (2013)[5], patient 6 (2018)[1]
429	g.157100452_157100461del	c.1389_1398del	p.(Ala464Serfs*35)	*de novo*	Targeted diagnostics			Yes	Aggregated	No		No		patient 124[1]
574	g.157100455_157100465del	c.1392_1402del	p.(Gln467Argfs*64)	unknown	Whole exome sequencing			No				No		
098	g.15100531_15100535del	c.1468_1472del	p.(Trp490Glyfs*43)	*de novo*	Whole genome sequencing			Yes	Yes	Yes		No		Patient # 10[9]
195	g.157100603C>T	c.1540C>T	p.(Gln514*)	unknown	Whole exome sequencing			No				No		
475	g.157100603C>T	c.1540C>T	p.(Gln514*)	*de novo*	Targeted diagnostics			No			Yes	Yes	Yes	
314	g.157100606G>A	c.1542+1G>A	r.(spl?)	*de novo*	Targeted diagnostics			Yes	Yes	Yes	Yes	No		patient 15 (2013)[5], patient 4 (2018)[1]
377	g.157150439C>T	c.1621C>T	p.(Gln541*)	unknown	Whole exome sequencing			No				Yes	Yes	
543	g.157177970_157432661del	c.1738-14778_2371+966del	p.(Asp580Valfs*54)	*de novo*	Array			No			Yes	Yes	Yes	
409	g.157222612del	c.1879del	p.(Ala627Argfs*41)	*de novo*	Whole exome sequencing			No			Yes	Yes	Yes	
565	g.157222630C>T	c.1897C>T	p.(Gln633*)	*de novo*	Whole exome sequencing			No			Yes	Yes	Yes	
192	g.157247195_157443054del	c.1927-9405_2372-11108del	p.(Asp643Valfs*54)	*de novo*	Array			No			Yes	No		
129	Breakpoint: chr6:157292076_157292079	c.2037+35366_2037+35369	46,XY,t(1;6)(p31;q25)	*de novo*	Karyotyping + mate pair sequencing			Yes	Yes	Yes		No		patient 1 (2012)[2], patient 71 (2018)[10]
389	g.157405795G>A	c.2038-1G>A	r.(spl?)	*de novo*	Whole exome sequencing			No				No		
054	g.157405927_157405946del	c.2169_2188del	p.(His723Glnfs*33)	*de novo*	Whole exome sequencing			Yes	Summier	Yes	Yes	Yes	No	case 2 (2016)[11], PED1306.1 (2018)[12]
122	g.157405994_157405995delinsGAGC	c.2236_2237delinsGAGC	p.(Ser746Glufs*37)	*de novo*	Whole exome sequencing			No				No		
349	g.157431642C>G	c.2318C>G	p.(Ser773*)	*de novo*	Whole exome sequencing			Yes	Aggregated	Yes	Yes	Yes	Yes	patient 84[1]
211	g.157431697T>C	c.2371+2T>C	r.(spl?)	unknown	Whole exome sequencing			Yes	Summier	Supplemented		No		Case 9[7]
525	g.157431700G>A	c.2371+5G>A	r.(spl?)	*de novo*	Whole exome sequencing			No			Yes	Yes	Yes	
375	g.157454309dup	c.2519dup	p.(Tyr840*)	*de novo*	Whole exome sequencing			No			Yes	Yes	Yes	
590	g.157454309dup	c.2519dup	p.(Tyr840*)	unknown	Targeted diagnostics			No			Yes	Yes	Yes	
451	g.157469758G>T	c.2552G>T	p.(Gly851Val)/r.(spl?)	*de novo*	Whole exome sequencing			No			Yes	No		
080	g.157469761_157470088del	c.2555_2879+3del	p.(Asn852Lysfs*7)	*de novo*	Targeted diagnostics			Yes	Summier	Yes		Yes	Yes	case 1[13]
343	g.157469898C>T	c.2692C>T	p.(Arg898*)	*de novo*	Whole exome sequencing			Yes	Aggregated	Yes	Yes	Yes	Yes	patient 125[1]
380	g.157167077dup	c.2917dup	p.(Met973Asnfs*16)	unknown	Targeted diagnostics			Yes	Aggregated	Yes	Yes	Yes	No	patient 128[1]
544	g.157488272dup	c.2978dup	p.(Ala994Glyfs*28)	*de novo*	Targeted diagnostics			No			Yes	Yes	Yes	
084	g.157495038_157495332del	c.3026-104_3135+81del	p.(Ala1009Glufs*21)	*de novo*	Targeted diagnostics			Yes	Summier	Yes		No		case 5[13]
467	g.157495193del	c.3077del	p.(Leu1026Argfs*104)	unknown	Whole genome sequencing			No			Yes	No		
342	g.157502190C>T	c.3223C>T	p.(Arg1075*)	unknown	Whole exome sequencing			Yes	Aggregated	Yes	Yes	Yes	Yes	patient 126[1]
373	g.157502190C>T	c.3223C>T	p.(Arg1075*)	*de novo*	Targeted diagnostics			Yes	Yes	Yes	Yes	Yes	Yes	subject 2 (2012)[6], patient 102 (2013)[5], patient 37 (2018)[1]
218	g.157502195C>G	c.3228C>G	p.(Tyr1076*)	unknown	Whole exome sequencing			No				No		
257	g.157502271C>T	c.3304C>T	p.(Arg1102*)	*de novo*	Whole exome sequencing			Yes	Aggregated	Yes	Yes	Yes	No	Leiden_D1.12.04221 (2017)[14], patient 12 (2018)[1]
680	g.157502271C>T	c.3304C>T	p.(Arg1102*)	*de novo*	Targeted diagnostics			Yes	Yes	Yes	Yes	Yes, published		patient 5[8]
534	g.157505364G>T	c.3346-1G>T	r.(spl?)	unknown	Whole exome sequencing			No			Yes	No		
414	g.157505452_157505453insTA	c.3433_3434insTA	p.(Tyr1145Leufs*67)	*de novo*	Whole exome sequencing			Yes	Yes	Yes	Yes	No, published (without eyes)		patient 1 (2016)[15], patient 103 (2018)[1]
134	g.157505497G>T	c.3478G>T	p.(Glu1160*)	unknown	Targeted diagnostics			No				Yes	Yes	
025	g.157510805dup	c.3580dup	p.(Thr1196Asnfs*16)	*de novo*	Targeted diagnostics			Yes	Summier	Yes		No		14393.p1[3]
472	g.157510811C>T	c.3586C>T	p.(Gln1196*)	*de novo*	Whole exome sequencing			No				No		
156	g.157511214_157511215del	c.3732_3733del	p.(Ser1244Argfs*32)	*de novo*	Whole exome sequencing			No				No		
256	g.157511328dup	c.3846dupT	p.(Gly1283Trpfs*38)	unknown	Targeted diagnostics			Yes	Yes	Yes	Yes	Yes	Yes	patient 10 (2013)[5], patient 1 (2018)[1]
587	g.157520028_157520032del	c.4097_4101del	p.(Tyr1366Serfs*91)	*de novo*	Whole exome sequencing			No			Yes	No		
119	g.157520041G>A	c.4110G>A	p.(Pro1370=)/r.(spl?)	*de novo*	Whole exome sequencing			No				No		
101	g.157521916C>G	c.4188C>G	p.(Tyr1396*)	not in mother	Whole genome sequencing			Yes	Aggregated	Supplemented	Yes	Yes	Yes	patient 30[4]
328	g.157522176_157522177ins14	c.4448_4449ins14	p.(Pro1489Leufs*10)	unknown	Targeted diagnostics			Yes	Yes	Yes	Yes	Yes	Yes	patient 51 (2013)[5], patient 34 (2018)[1]

Supplementary Table 1: ***continued***

Patient ID	Genomic location	cDNA	Protein change	Inheritance	Technique	Parent/via child identified	Mosaic	Published?	Published Phenotype data	Data updated	Parental questionnaire	Facial photo available	Consent publication	Published patient number
348	g.157522346_157522356del	c.4619_4628del	p.(Gln1541Argfs*35)	*de novo*	Targeted diagnostics			Yes	Yes	Yes		Yes, published		subject 3 (2012)[6], patient 103 (2013)[5], patient 49 (2018)[1]
293	g.157522351_157522369dup	c.4623_4641dup	p.(Asn1548Aspfs*94)	*de novo*	Whole exome sequencing			No			Yes	Yes	No	
033	g.157522406del	c.4678del	p.(Gln1560Serfs*19)	unknown	Whole exome sequencing			No				Yes	Yes	
473	g.157522575C>G	c.4847C>G	p.(Ser1616*)	unknown	Whole exome sequencing			Yes	Aggregated	Yes	Yes	Yes	Yes	patient 30[1]
340	g.157522598C>T	c.4870C>T	p.(Arg1624*)	unknown	Whole exome sequencing			No			Yes	Yes	Yes	
203	g.157525128C>T	c.5023C>T	p.(Gln1675*)	*de novo*	Whole exome sequencing			No			Yes	Yes	Yes	
157	g.157527299A>C	c.5026-2A>C	r.(spl?)	*de novo*	Targeted diagnostics			No			Yes	Yes	Yes	
093	g.157527378T>A	c.5103T>A	p.(Tyr1701*)	*de novo*	not reported			Yes	Yes	Yes	Yes	Yes	Yes	Patient # 5[9]
575	g.157527532G>T	c.5257G>T	p.(Glu1753*)	*de novo*	Targeted diagnostics			Yes	Aggregated	Yes		Yes	Yes	patient 28[1]
474	g.157527534_157527535dup	c.5259_5260dup	p.(Lys1754Argfs*13)	unknown	Targeted diagnostics			Yes	Yes	No		Yes, published		Subject 1[16]
395	g.157527598_157527599dup	c.5324_5325insAA	p.(Glu1776Argfs*15)	*de novo*	Whole exome sequencing			No			Yes	Yes	Yes	
344	g.157527604A>T	c.5329A>T	p.(Lys1777*)	*de novo*	Whole exome sequencing			Yes	Aggregated	Yes	Yes	Yes	Yes	subject 1 (2012)[6], patient 48 (2018)[1]
175	g.157527679C>T	c.5404C>T	p.(Arg1802*)	unknown	not reported			No			Yes	Yes	No	
263	g.157527901dup	c.5626dup	p.(Thr1876Asnfs*4)	*de novo*	Whole exome sequencing			Yes	Aggregated	Yes	Yes	Yes	Yes	patient 136[1]
661	g.157527822dup	c.5547dup	p.(Ser1851Lysfs*5)	unknown	not reported			No				No		
419	g.157528051C>T	c.5776C>T	p.(Arg1926*)	*de novo*	Whole exome sequencing			Yes	Yes	Yes	Yes	No, published (without eyes)		patient 1[17]
017	g.157528225G>T	c.5950G>T	p.(Glu1984*)	not in mother	Targeted diagnostics			No				No		
096	g.157528243C>T	c.5968C>T	p.(Arg1990*)	*de novo*	Targeted diagnostics			Yes	Yes	Yes	Yes	Yes	Yes	Patient # 8[9]
506	g.157528268_157528271del	c.5993_5996del	p.(Glu1998Glyfs*22)	*De novo*	Targeted diagnostics			No			Yes	Yes	Yes	
210	g.157528313G>A	c.6038G>A	p.(Trp2013*)	*de novo*	Whole exome sequencing			No			Yes	Yes	No	
456	g.157528439G>A	c.6164G>A	p.(Trp2055*)	*de novo*	Whole exome sequencing			No				No		
324	g.157528508del	c.6233del	p.(Pro2078Leufs*21)	*de novo*	Targeted diagnostics			Yes	Yes	No		Yes, published (supplementary)		patient 39 (2013)[5], patient 27 (2018)[1]
346	g.157528513A>T	c.6238A>T	p.(Arg2080*)	*de novo*	Whole exome sequencing			Yes	Aggregated	No		No		patient 105[1]
066	g.157528597C>T	c.6322C>T	p.(Gln2108*)	*de novo*	Exome (variant in child)	Yes	Yes	Yes	Summier	Yes		Yes	No	Case 12[7]
468	g.157528657C>T	c.6382C>T	p.(Arg2128*)	*de novo*	Whole exome sequencing			No				No		
181	g.157528719_157528721delinsTGTGCAGA AAGGGCTGTGCAGAA	c.6444_6446delinsTGTGCAGAAA	p.(Ile2149Valfs*18)	*de novo*	Whole exome sequencing			No			Yes	Yes	No	
679	g.157528738_157528748del	c.6463_6473del	p.(Ser2155Leufs*33)	*de novo*	Targeted diagnostics			Yes	Yes	Yes	Yes	Yes, published		patient 4[8]
190	g.157529008C>T	c.6733C>T	p.(Gln2245*)	*de novo*	Whole exome sequencing			No			Yes	Yes	No	

All variants have been uploaded in the LOVD database: www.lovd.nl/ARID1B

References

1. van der Sluijs PJ, Jansen S, Vergano SA, et al. The ARID1B spectrum in 143 patients: from nonsyndromic intellectual disability to Coffin–Siris syndrome. *Genetics in Medicine*. 2018/10/22 2018;doi:10.1038/s41436-018-0330-z
2. Halgren C, Kjaergaard S, Bak M, et al. Corpus callosum abnormalities, intellectual disability, speech impairment, and autism in patients with haploinsufficiency of ARID1B. *Clin Genet*. Sep 2012;82(3):248-55. doi:10.1111/j.1399-0004.2011.01755.x
3. O'Roak BJ, Vives L, Fu W, et al. Multiplex targeted sequencing identifies recurrently mutated genes in autism spectrum disorders. *Science*. Dec 21 2012;338(6114):1619-22. doi:10.1126/science.1227764
4. van der Sluijs PJ, Joosten M, Alby C, et al. Discovering a new part of the phenotypic spectrum of Coffin-Siris syndrome in a fetal cohort. *Genet Med*. May 17 2022;doi:10.1016/j.gim.2022.04.010
5. Santen GW, Aten E, Vulto-van Silfhout AT, et al. Coffin-Siris syndrome and the BAF complex: genotype-phenotype study in 63 patients. *Hum Mutat*. Nov 2013;34(11):1519-28. doi:10.1002/humu.22394
6. Santen GW, Aten E, Sun Y, et al. Mutations in SWI/SNF chromatin remodeling complex gene ARID1B cause Coffin-Siris syndrome. *Nat Genet*. Mar 18 2012;44(4):379-80. doi:10.1038/ng.2217
7. van der Sluijs PJ, Alders M, Dingemans AJM, et al. A Case Series of Familial ARID1B Variants Illustrating Variable Expression and Suggestions to Update the ACMG Criteria. *Genes*. 2021;12(8):1275.
8. Hoyer J, Ekici AB, Endele S, et al. Haploinsufficiency of ARID1B, a member of the SWI/SNF-a chromatin-remodeling complex, is a frequent cause of intellectual disability. *Am J Hum Genet*. Mar 09 2012;90(3):565-72. doi:10.1016/j.ajhg.2012.02.007
9. Mignot C, Moutard ML, Rastetter A, et al. ARID1B mutations are the major genetic cause of corpus callosum anomalies in patients with intellectual disability. *Brain*. Jul 29 2016;139(11):e64. doi:10.1093/brain/aww181
10. Halgren C, Nielsen NM, Nazaryan-Petersen L, et al. Risks and Recommendations in Prenatally Detected De Novo Balanced Chromosomal Rearrangements from Assessment of Long-Term Outcomes. *Am J Hum Genet*. Jun 7 2018;102(6):1090-1103. doi:10.1016/j.ajhg.2018.04.005
11. Thevenon J, Duffourd Y, Masurel-Paulet A, et al. Diagnostic odyssey in severe neurodevelopmental disorders: toward clinical whole-exome sequencing as a first-line diagnostic test. *Clin Genet*. Jun 2016;89(6):700-7. doi:10.1111/cge.12732
12. Nambot S, Thevenon J, Kuentz P, et al. Clinical whole-exome sequencing for the diagnosis of rare disorders with congenital anomalies and/or intellectual disability: substantial interest of prospective annual reanalysis. *Genet Med*. Jun 2018;20(6):645-654. doi:10.1038/gim.2017.162
13. Gorokhova S, Mortreux J, Afenjar A, et al. Significant contribution of intragenic deletions to ARID1B mutation spectrum. *Genet Med*. May 20 2019;doi:10.1038/s41436-019-0546-6
14. Stessman HA, Xiong B, Coe BP, et al. Targeted sequencing identifies 91 neurodevelopmental-disorder risk genes with autism and developmental-disability biases. *Nat Genet*. Apr 2017;49(4):515-526. doi:10.1038/ng.3792
15. Sonmez FM, Uctepe E, Gunduz M, et al. Coffin-Siris syndrome with cafe-au-lait spots, obesity and hyperinsulinism caused by a mutation in the ARID1B gene. *Intractable Rare Dis Res*. Aug 2016;5(3):222-6. doi:10.5582/irdr.2014.01040
16. Maattanen L, Hietala M, Ignatius J, Arvio M. A 69-year-old woman with Coffin-Siris syndrome. *Am J Med Genet A*. Jul 28 2018;doi:10.1002/ajmg.a.38844
17. Uctepe E, Erguner B, Sonmez FM. Recurrence of ARID1B -related Coffin-Siris Syndrome by possible gonadal mosaicism. *Clin Dysmorphol*. Oct 1 2023;32(4):180-183. doi:10.1097/MCD.0000000000000473

Supplementary Figure 1

Supplementary Figure 1A) Facial photographs of *ARID1B* patients aged 0-4 years

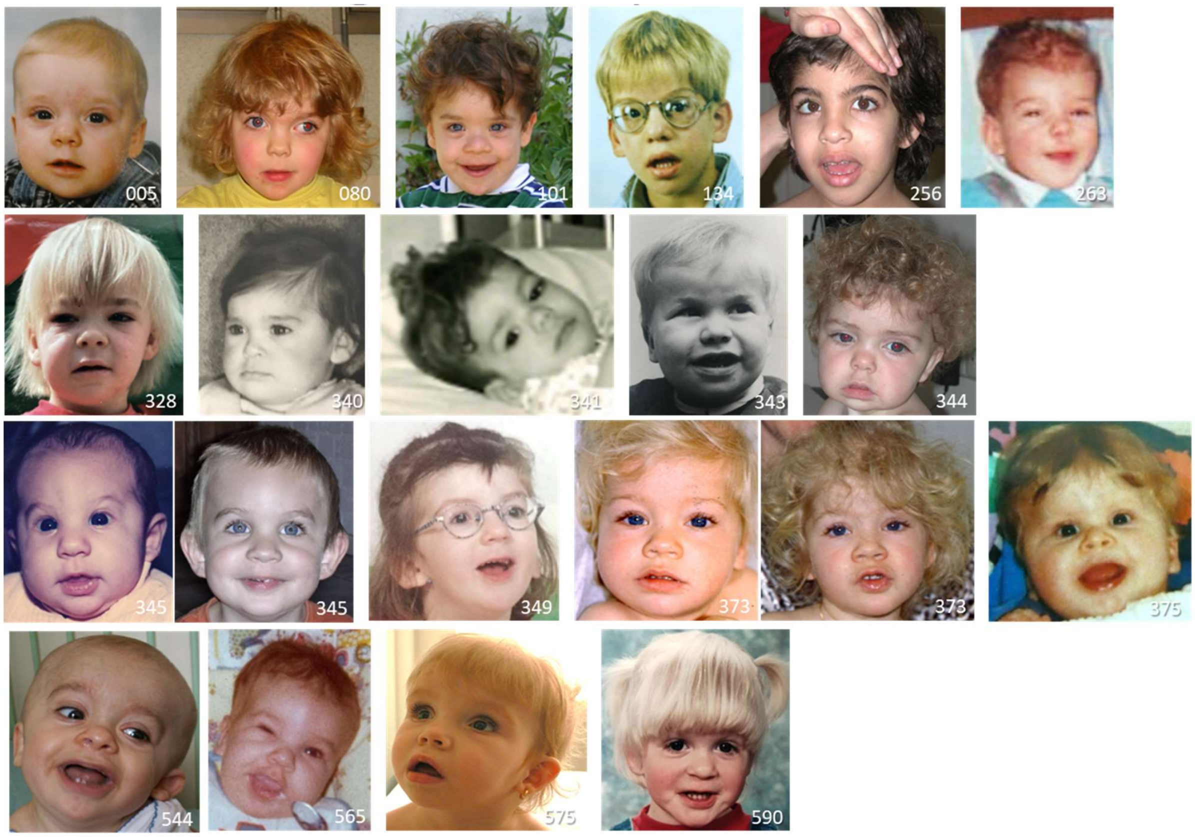

Supplementary Figure 1B) Facial photographs of *ARID1B* patients aged 5-10 years

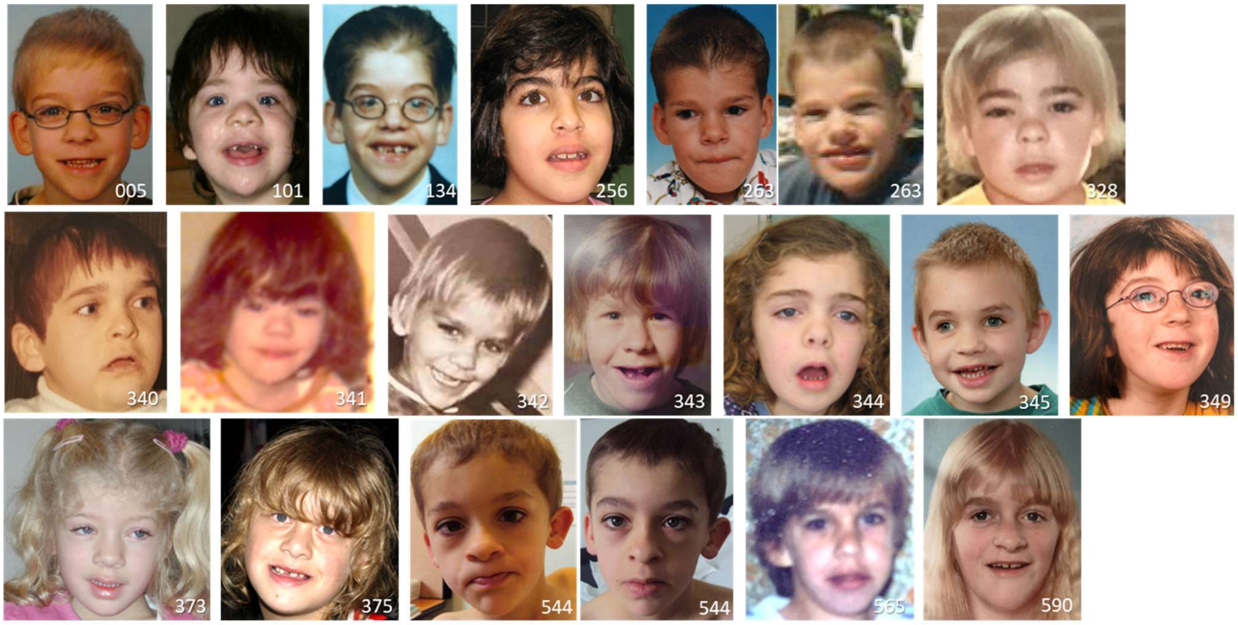

Supplementary Figure 1C) Facial photographs of *ARID1B* patients aged 11-17 years

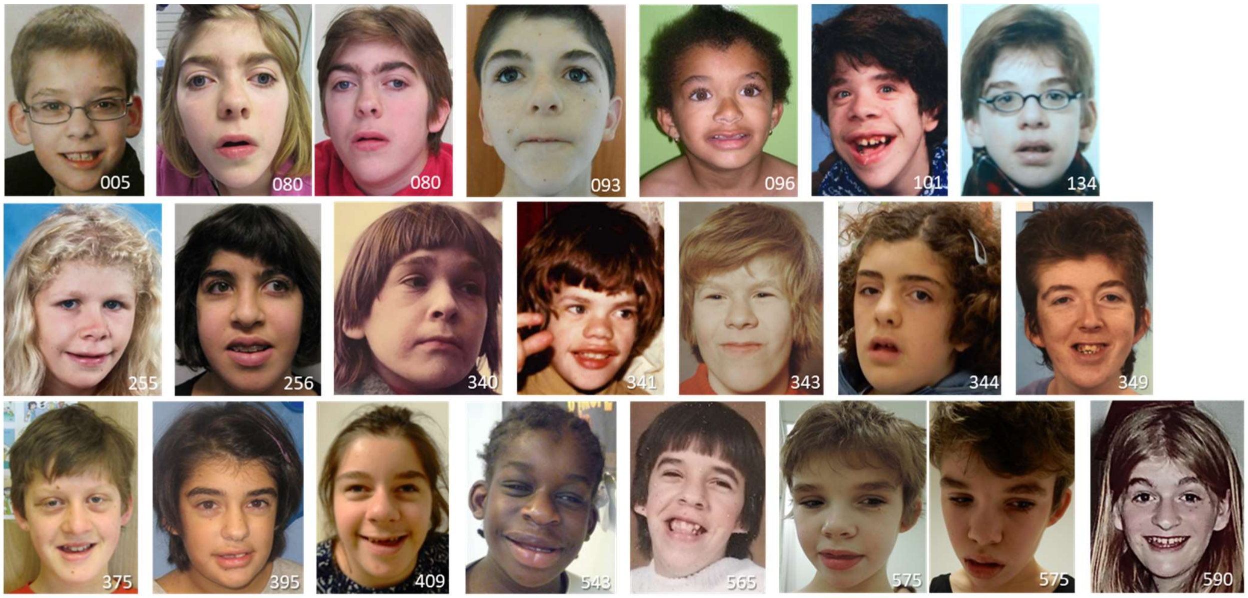

Supplementary Figure 1D) Facial photographs of *ARID1B* patients aged 18-25 years

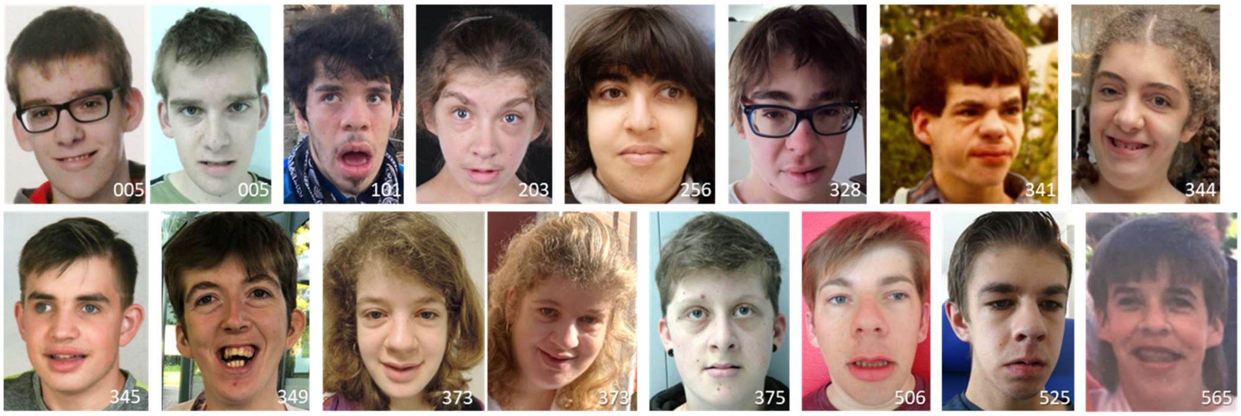

Supplementary Figure 1E) Facial photographs of *ARID1B* patients aged 26+ years

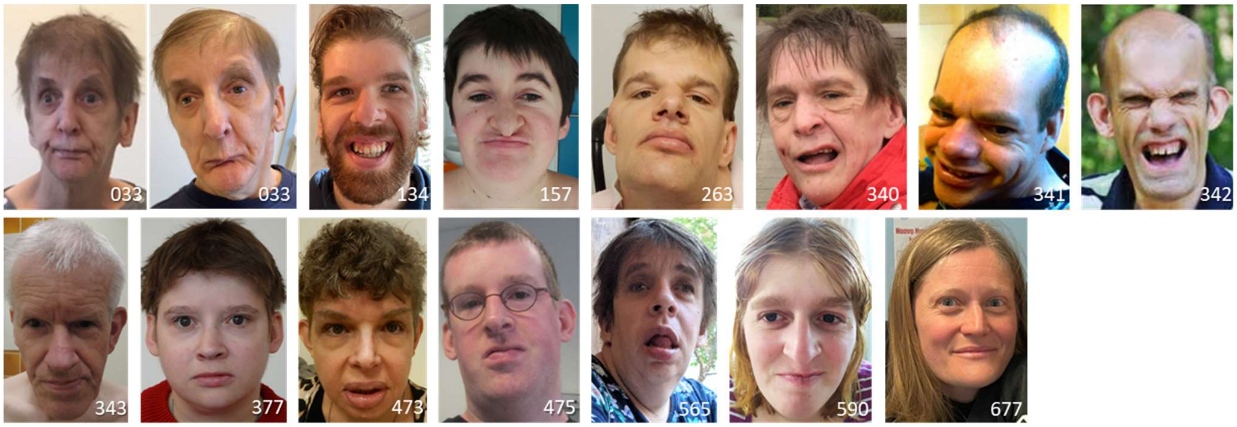

Supplementary Figure 2: DNA methylation clustering results of case 677 with NM_020732.3:c. c.63_73del p.(Glu22Glnfs*206)

Supplementary Figure 2A: Hierarchical clustering with heatmap of methylation data

Supplementary Figure 2B: MDS-plot

Supplementary Figure 2C: MVP-score

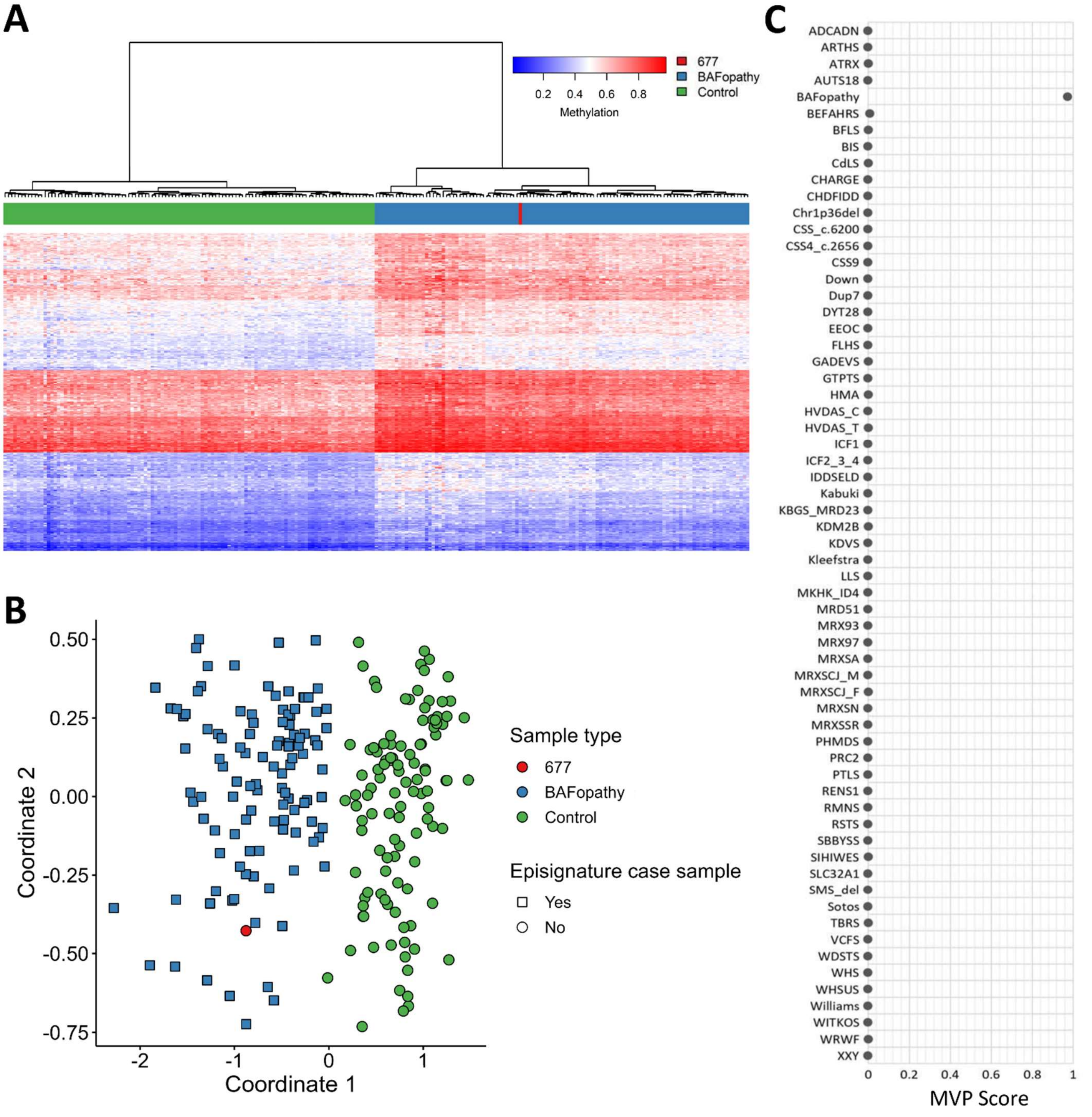

Supplementary Table 2: Clinical characteristics of *ARID1B* patients

	18+ (LoF)					LoF patients exon 1			LoF patients >exon 1			Mosaic patients		
Clinical features +	*n* = 85	pt affected	%	*p-value**	*Test**	*n* = 16	pt affected	%	*n* = 69	pt affected	%	*n* = 2	pt affected	%
Age (n; median; min-max)	85	22,3	(18-69)	0,64	T	16	24,0	(18,5-48,5)	69	22,0	(18-69)	2	36,0	(35-37)
Sex (female)	85	52	61%	0,78	Chi	16	9	56%	69	43	62%	2	1	50%
Died	85	2	2%	1,00	F	16	0	0%	69	2	3%	2	0	0%
Growth parameters & development														
Gestational age, weeks (mean; SD)	73	39,0	224%	0,19	T	11	38,1	275%	62	39,1	213%	1	40	-
Birthweight (<-2 SDS)	53	6	11%	0,58	F	7	0	0%	46	6	13%	0	0	-
Height at birth (<-2 SDS)	16	5	31%	1,00	F	2	0	0%	14	5	36%	0	0	-
OFC at birth (<-2 SDS)	18	1	6%	1,00	F	3	0	0%	15	1	7%	0	0	-
Age last measurements, years (n; median; min-max)	83	22,0	(4,2-69)	0,59	T	16	22,1	(11,8-40)	67	22,0	(4,2-69)	2	36,0	(35-37)
Weight (<-2 SDS)	55	0	0%	-	-	6	0	0%	49	0	0%	0	0	-
BMI >25 or overweight	70	39	56%	1,00	F	10	6	60%	60	33	55%	1	1	100%
Lenght (<-2 SDS)	69	37	54%	0,49	F	9	6	67%	60	31	52%	2	1	50%
OFC (<-2 SDS)	55	3	5%	1,00	F	9	0	0%	46	3	7%	1	0	0%
Motor delay	78	72	92%	0,01	F	14	10	71%	64	62	97%	2	0	0%
Motor skills gross, delayed	78	59	76%	0,31	F	14	9	64%	64	50	78%	2	0	0%
Motor skills fine, delayed	78	51	65%	0,07	F	14	6	43%	64	45	70%	2	0	0%
Speech, delayed	81	79	98%	0,35	F	15	14	93%	66	65	98%	2	0	0%
Sleeping problems	59	21	36%	0,24	F	8	1	13%	51	20	39%	1	0	0%
Obstructive sleep apnea	85	1	1%	1,00	F	16	0	0%	69	1	1%	2	0	0%
Laryngomalacia	53	5	9%	1,00	F	10	1	10%	43	4	9%	2	0	0%
Feeding difficulties	81	53	65%	0,03	Chi	15	6	40%	66	47	71%	2	0	0%
Duration of feeding problems	42			0,17	F	5			37			0		
Brief		17	40%				3	60%		14	38%		0	-
Several years		16	38%				0	0%		16	43%		0	-
Ongoing		9	21%				2	40%		7	19%		0	-
Recurrent infections	71	28	39%	0,75	F	12	4	33%	59	24	41%	1	0	0%
Upper airway tract	71	3	4%	1,00	F			0%		3	5%	1	0	0%
Lower airway tract	71	2	3%	1,00	F			0%		2	3%	1	0	0%
ENT infections	71	12	17%	0,68	F			8%		11	19%	1	0	0%
Otitis media	71	10	14%	0,67	F			17%		8	14%	1	0	0%
Urinary tract	71	6	8%	1,00	F			8%		5	8%	1	0	0%
Neurological features														
IQ (median; (min-max))	32	55	(20-80)	0,48	T	4	58-5	(54-65)	28	55,0	(20-80)	0	-	-
Intellectual disability	85	79	93%	0,08	F	16	13	81%	69	66	96%	2	0	0%
Borderline	0	6	7%				3	19%		3	4%		1	50%
Mild	0	24	28%				6	38%		18	26%		0	0%
Mild-moderate	-	-	-	-	-	-	-	-	-	-	-	-	-	-
Moderate	0	36	42%				5	31%		31	45%		0	0%
Moderate-severe	-	-	-	-	-	-	-	-	-	-	-	-	-	-
Severe	0	17	20%				2	13%		15	22%		0	0%
Profound			0%				0	0%		2	3%		0	0%
Hypotonia	74	57	77%	0,13	F	12	7	58%	62	50	81%	2	0	0%
Seizures	81	38	47%	0,67	F	14	5	36%	67	33	49%	2	0	0%
No seizures, but abnormal EEG		6	7%				1	7%		5	7%		0	0%
Still experiencing seizures	20	3	15%	0,15	F	1	1	100%	19	2	11%	0	0	-
Loss of skills	64	16	25%	0,45	F	10	0	0%	54	16	30%	2	0	0%
Motoric	0	7	11%				0	0%		7	13%		0	0%
Speech	0	5	8%				0	0%		5	9%		0	0%
Unspecified	0	5	8%				0	0%		5	9%		0	0%
Agenesis of the corpus callosum	58	27	47%	0,94	F	8	4	50%	50	23	46%	2	0	0%
Partial/hypoplasia		18	31%				3	38%		15	30%		0	0%
Brain abnormality	67	37	55%	1,00	F	10	6	60%	57	31	54%	2	1	50%
MRI performed	78	66	85%	0,05	Chi	15	10	67%	63	56	89%	1	1	100%
Vision and hearing impairments														
Vision impaired	82	68	83%	1,00	F	15	13	87%	67	55	82%	2	0	0%
Myopia	60	47	78%	1,00	F	12	10	83%	48	37	77%	0	0	-
Hypermetropia	53	14	26%	0,09	F	8	0	0%	45	14	31%	0	0	-
Cataract	43	3	7%	1,00	F	7	0	0%	36	3	8%	1	0	0%
Hearing loss	79	22	28%	1,00	F	15	4	27%	64	18	28%	2	0	0%
Hearing loss, conductive	79	11	14%	1,00	F			13%		9	14%	2	0	0%
Hearing loss, perceptive	79	6	8%	0,59	F			0%		6	9%	2	0	0%
Eartubes	56	23	41%	1,00	F	11	4	36%	45	19	42%	2	0	0%
Hearing aid	17	6	35%	1,00	F	3	1	33%	14	5	36%	0	0	-
Musculoskeletal anomalies														
Orthopedic anomalies (scoliosis+patella+pes pedes)	85	52	61%	0,16	Chi	16	7	44%	69	45	65%	2	1	50%
Scoliosis	82	25	30%	1,00	F	15	4	27%	67	21	31%	2	1	50%
Degree scoliosis (median (min-max), SD)	3	46	(37-75)	-	-	0	-	-	3	46	(37-75)	0	-	-
Operation scoliosis needed	22	7	32%	0,52	F	3	0	0%	19	7	37%	1	0	0%
Pes planus	46	31	67%	0,65	F	6	5	83%	40	26	65%	2	1	50%
Patella luxation	45	12	27%	0,84	F	8	1	13%	37	11	30%	1	0	0%
Recurrent	12	8	67%			1	1	100%	11	7	64%		0	
Pectus, excavatum	80	2	3%	1,00	F	15	0	0%	65	2	3%	1	0	0%
Primary dentition, delayed	47	11	23%	0,66	F	9	1	11%	38	10	26%	2	0	0%
Permanent dentition, delayed	47	19	40%	0,28	F	9	2	22%	38	17	45%	2	0	0%
Widely spaced teeth	47	7	15%	1,00	F	9	1	11%	38	6	16%	2	0	0%
Abnormal dentition	36	25	69%	1,00	F	4	3	75%	32	22	69%	2	1	50%
Dental surgeon operation/treated by a dental surgeon	34	17	50%	0,38	F	5	1	20%	29	16	55%	0	-	-
Joint laxity	46	23	50%	0,24	F	8	2	25%	38	21	55%	2	0	0%
Early arthritis	33	2	6%	1,00	F	7	0	0%	26	2	8%	1	0	0%
Clinodactyly	65	8	12%	0,63	F	12	2	17%	53	6	11%	2	0	0%
Brachydactyly fifth finger	65	16	25%	1,00	F	12	3	25%	53	13	25%	2	0	0%
Small nails	66	25	38%	1,00	Chi	13	6	46%	53	19	36%	2	0	0%
Which nails, 5th finger and/or toe	66	19	29%	0,17	Chi	13	6	46%	53	13	25%	2	0	0%
Intestinal														
Inguinal hernia	53	4	8%	0,54	F	9	1	11%	44	3	7%	1	0	0%
Intestinal problems	73	31	42%	0,54	F	13	4	31%	60	27	45%	1	0	0%
Constipation	73	20	27%	0,10	F	13	1	8%	60	19	32%	1	0	0%
Gastroesophageal reflux	73	8	11%	0,63	F	13	2	15%	60	6	10%	1	0	0%
Diarrhea	73	0	0%	-	-	13	0	0%	60	0	0%	1	0	0%
Pyloric Stenosis	73	0	0%	-	-	13		0%	60	0	0%	1	0	0%
Umbilical hernia	73	3	4%	0,08	F	13	2	15%	60	1	2%	1	0	0%

	18+ (LoF)					LoF patients exon 1			LoF patients >exon 1			Mosaic patients		
Clinical features +	*n* = 85	pt affected	%	*p-value**	*Test**	*n* = 16	pt affected	%	*n* = 69	pt affected	%	*n* = 2	pt affected	%
Cardiac & urogenital anomalies														
Cardiac anomalies	65	9	14%	1,00	F	12	1	8%	53	8	15%	1	0	0%
ASD	65	4	6%	1,00	F	12	0	0%	53	4	8%	1	0	0%
VSD	65	0	0%	-	-	12	0	0%	53	0	0%	1	0	0%
Aortic valve abnormality	65	2	3%	0,34	F	12	1	8%	53	1	2%	1	0	0%
Mitralis insufficiency	65	1	2%	1,00	F	12	0	0%	53	1	2%	1	0	0%
Renal anomalies	42	18	43%	0,01	F	6	0	0%	36	18	50%	0	-	-
Hydronephrotic kidney	42	4	10%	1,00	F	6	0	0%	36	4	11%	0	-	-
Nephrolithiasis	42	9	21%	0,31	F	6	0	0%	36	9	25%	0	-	-
Renal sonography, abnormal	43	18	42%			6	0	0%	37	18	49%	0	-	-
Age identification first renal stone (nr, median, min-max)	5	31	(7-59)	-	-	0	-	-	5	31	(7-59)	0	-	-
Cryptorchidism	30	18	60%	0,66	F	6	3	50%	24	15	63%	1	0	0%
Endocrinological abnormalities														
Diabetes mellitus	54	6	11%	1,00	F	8	1	13%	46	5	11%	0	-	-
Type 2 diabetes mellitus	54	6	11%	1,00	F	8	1	13%	46	5	11%	0	-	-
Hypothyroidism	54	8	15%	1,00	F	8	1	13%	46	7	15%	0	-	-
Growth hormone deficiency	54	1	2%	1,00	F	8	0	0%	46	1	2%	0	-	-
Other														
Anemia	54	3	6%	1,00	F	8	0	0%	46	3	7%	0	-	-
Elevated cholesterol	54	4	7%	1,00	F	8	0	0%	46	4	9%	0	-	-
Hypertension	35	6	17%	0,56	F	5	0	0%	30	6	20%	0	-	-
Behavioral abnormalities	80	68	85%	0,40	F	13	10	77%	67	58	87%	2	1	50%
Hyperactivity	75	5	7%	0,59	F	13	0	0%	62	5	8%	2	0	0%
High pain threshold	53	34	64%	0,26	F	9	4	44%	44	30	68%	1	0	0%
Psychiatric disorders														
ADHD	80	7	9%	1,00	F	13	1	8%	67	6	9%	2	1	50%
Autistic traits	80	21	26%	0,50	F	13	2	15%	67	19	28%	2	0	0%
Autism	80	25	31%	0,75	F	13	3	23%	67	22	33%	2	0	0%
Age autism diagnosis (nr, median, min-max)	21	7	(0-25)	0,89	MW	3	12	(3-12)	18	7	(0-25)	0	-	-
Auto-mutilation	80	15	19%	0,11	F	13	0	0%	67	15	22%	2	0	0%
Malignancies	73	1	1%	1,00	F	12	0	0%	61	1	2%	2	0	0%
Lifestyle														
Daycare	49	32	65%	0,15	F	7	4	57%	42	28	67%	0	-	-
Regular	0	11	22%				3	43%		8	19%			
Special	0	21	43%				1	14%		20	48%			
Primary education	59	59	100%	0,15	F	8	8	100%	51	51	100%	0	-	-
Regular		1	2%				1	13%		0	0%			
Special		41	69%				7	88%		34	67%			
Secondary education	45	33	73%	0,38	F	7	6	86%	38	27	71%	0	-	-
Regular	0	30	67%				1	14%		2	5%			
Special	0	0	0%				5	71%		25	66%			
Living situation	57			0,22	F	8		0%	49		0%	0	-	-
At home/with parents		38	67%				6	75%		32	65%	0	-	-
Independently guided/assisted living		5	9%				1	13%		4	8%	0	-	-
Residential group (>residents/caretaker)		11	19%				0	0%		11	22%	0	-	-
Residential group (1 on 1 guidance)		3	5%				1	13%		2	4%	0	-	-
Medication	65	48	74%	0,69	F	9	6	67%	56	42	75%	2	2	100%
Anti-epileptics	65	15	23%	1,00	F	9	2	22%	56	13	23%	2	0	0%
Anti-depressants	65	8	12%	0,31	F	9	2	22%	56	6	11%	2	1	50%
Anti-psychotics	65	8	12%	0,59	F	9	0	0%	56	8	14%	2	0	0%
Diuretics/Anti-hypertensives	65	8	12%	1,00	F	9	1	11%	56	7	13%	2	1	50%
Amphetamines	65	3	5%	1,00	F	9	0	0%	56	3	5%	2	0	0%
Anti-diabetics	65	5	8%	1,00	F	9	0	0%	56	5	9%	2	0	0%
Hypo-/hyperthyroidism medication	65	5	8%	0,14	F	9	2	22%	56	3	5%	2	1	50%
Laxatives	65	10	15%	0,33	F	9	0	0%	56	10	18%	2	0	0%
PPI	65	7	11%	0,58	F	9	0	0%	56	7	13%	2	0	0%
Other	65	28	43%	0,07	F	9	1	11%	56	27	48%	2	1	50%

+ the total number of a feature can differ from the sum of subcategories, because in some cases it was possible to answer with more than 1 option or to report the existence of a feature without specifying.

* Groups compared are patients with a pathogenic variant in exon 1 versus patients with an exon 2-20 variant or a deletion in *ARID1B*.

Abbreviations

SDS: Standard Deviation Score
BMI: Body Mass Index
OFC: OccipitoFrontal Circumference
SD: Standard Deviation
ENT: Ear Nose Throat
EEG: ElectroEncephaloGraphy
ASD: Atrial Septal Defect
VSD: Ventricular Septal Defect
ADHD: Attention Deficit Hyperactivity Disorder
LoF: Loss of Function variants
F: Fisher's exact
Chi: Chi-square
T: T-test
A: ANOVA
KW: Kruskal-Wallis
MW: Mann-Whitney U

Supplementary Figure 3

Supplementary Figure 3A) Cumulative distribution: toilet trained urine, *n*=45

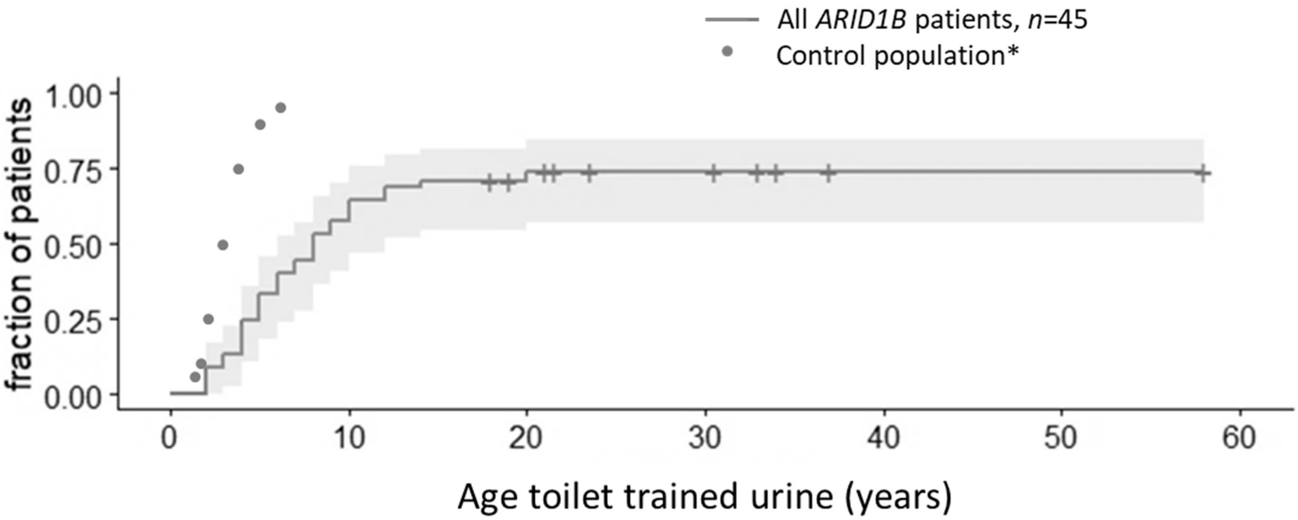

Supplementary Figure 3B) Histogram Age onset puberty, *n*=39

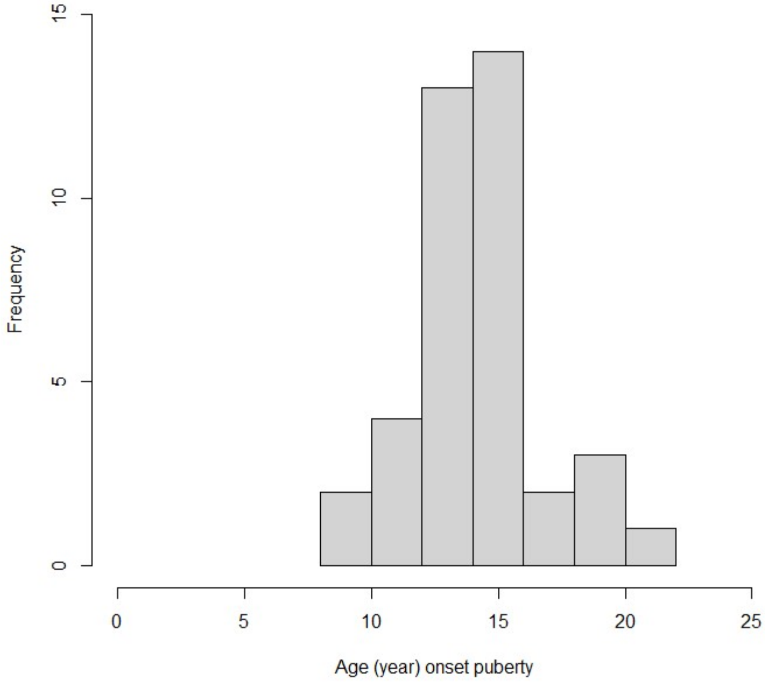

Supplementary Figure 3C) Histogram Myopia severity, *n*=27

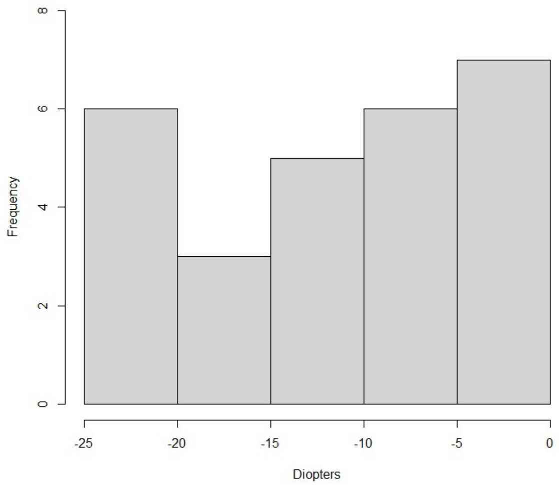

Supplementary Table 3: Activities of daily living: Parent reported outcomes for their adult children

	18+ (LoF)					LoF patients exon 1			LoF patients >exon 1			Mosaic patients		
	n = 85	pt affected	%	Test*	p-value*	*n* = 16	pt affected	%	*n* = 69	pt affected	%	*n* = 2	pt affected	%
Can your child make his/her own bed?	49			F	0,48	7			42			0		
No		8	16,3%				0	0,0%		8	19,0%		-	
With help		26	53%				4	57%		22	52%		-	
Independently		15	31%				3	43%		12	29%		-	
Can your child clean up, do light housework?	51			F	0,73	7			44			0		
No		7	13,7%				0	0,0%		7	15,9%		-	
With help		30	59%				5	71%		25	57%		-	
Independently		14	27%				2	29%		12	27%		-	
Can your child do the groceries?	50			F	0,06	7			43			0		
No		18	36,0%				0	0,0%		18	41,9%		-	
With help		17	34%				3	43%		14	33%		-	
Independently		15	30%				4	57%		11	26%		-	
Can your child replace a lamp, tighten screw?	50			F	0,00	7			43			0		
No		27	54,0%				0	0,0%		27	62,8%		-	
With help		16	32%				5	71%		11	26%		-	
Independently		7	14%				2	29%		5	12%		-	
Can your child do the laundry?	49			F	0,03	7			42			0		
No		18	36,7%				0	0,0%		18	42,9%		-	
With help		23	47%				4	57%		19	45%		-	
Independently		8	16%				3	43%		5	12%		-	
Can your child take a bath or shower?	51			F	0,05	7			44			0		
No		4	7,8%				0	0,0%		4	9,1%		-	
With help		26	51%				1	14%		25	57%		-	
Independently		21	41%				6	86%		15	34%		-	
Can your child brush his/her teeth and comb his/her hair?	51			F	0,19	7			44			0		
No		8	15,7%				0	0,0%		8	18,2%		-	
With help		24	47%				2	29%		22	50%		-	
Independently		19	37%				5	71%		14	32%		-	
Can your child dress and undress him/herself?	51			F	0,26	7			44			0		
No		3	5,9%				0	0,0%		3	6,8%		-	
With help		11	22%				0	0%		11	25%		-	
Independently		37	73%				7	100%		30	68%		-	
Can your child go to the toilet?	51			F	0,74	7			44			0		
No		3	5,9%				0	0,0%		3	6,8%		-	
With help		8	16%				0	0%		8	18%		-	
Independently		40	78%				7	100%		33	75%		-	
Can your child make sandwiches?	51			F	0,16	7			44			0		
No		10	19,6%				0	0,0%		10	22,7%		-	
With help		15	29%				1	14%		14	32%		-	
Independently		26	51%				6	86%		20	45%		-	
Can your child fry an egg, make pancakes, or heat food in the microwave?	50			F	0,25	7			43			0		
No		18	36,0%				1	14,3%		17	39,5%		-	
With help		24	48%				4	57%		20	47%		-	
Independently		8	16%				2	29%		6	14%		-	
Can your child prepare dinner?	49			F	0,02	7			42			0		
No		27	55,1%				1	14,3%		26	61,9%		-	
With help		18	37%				4	57%		14	33%		-	
Independently		4	8%				2	29%		2	5%		-	
Can your child set and clear the table?	50			F	0,41	7			43			0		
No		6	12,0%				0	0,0%		6	14,0%		-	
With help		15	30%				1	14%		14	33%		-	
Independently		29	58%				6	86%		23	53%		-	
Can your child drink from a cup?	51			F	1,00	7			44			0		
No		2	3,9%				0	0,0%		2	4,5%		-	
With help		0	0%				0	0%		0	0%		-	
Independently		49	96%				7	100%		42	95%		-	
Can your child eat from a plate?	51			F	1,00	7			44			0		
No		1	2,0%				0	0,0%		1	2,3%		-	
With help		4	8%				0	0%		4	9%		-	
Independently		46	90%				7	100%		39	89%		-	
Can your child do the dishes or load the dishwasher?	47			F	0,64	7			40			0		
No		7	14,9%				0	0,0%		7	17,5%		-	
With help		14	30%				2	29%		12	30%		-	
Independently		26	55%				5	71%		21	53%		-	
Can your child handle money, pay in the store?	46			F	0,21	7			39			0		
No		24	52,2%				2	28,6%		22	56,4%		-	
With help		16	35%				3	43%		13	33%		-	
Independently		6	13%				2	29%		4	10%		-	
Can your child stay home alone for 30 minutes?	46			F	0,39	7			39			0		
No		16	34,8%				1	14,3%		15	38,5%		-	
Yes		30	65%				6	86%		24	62%		-	
Can your child travel alone by public transport?	45			F	0,30	7			38			0		
No		38	84,4%				5	71,4%		33	86,8%		-	
Yes		7	16%				2	29%		5	13%		-	

Abbreviations:
F: Fisher's exact
LoF: Loss of Function variants
* Groups compared are patients with a pathogenic variant in exon 1 versus patients with an exon 2-20 variant or a deletion in *ARID1B*

Supplementary Figure 4

Supplementary Figure 4A) Cumulative distribution: sitting, *n*=60

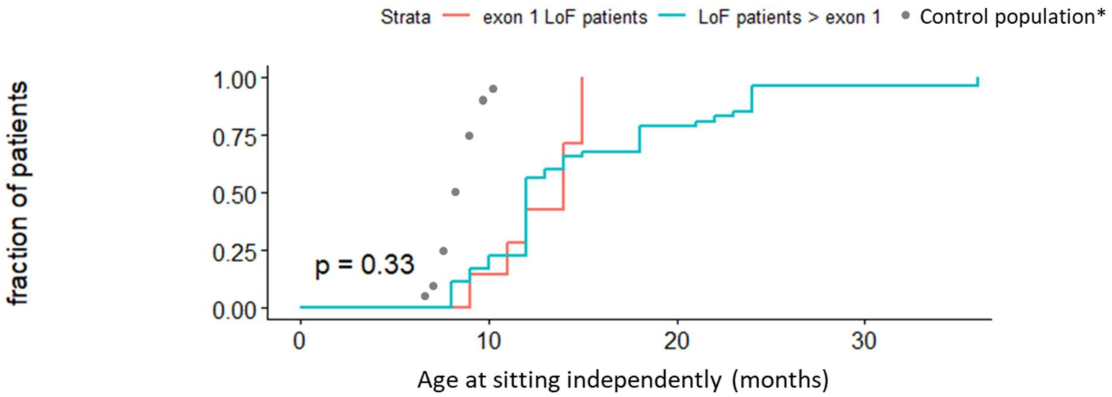

Supplementary Figure 4B) Cumulative distribution: walking, *n*=74

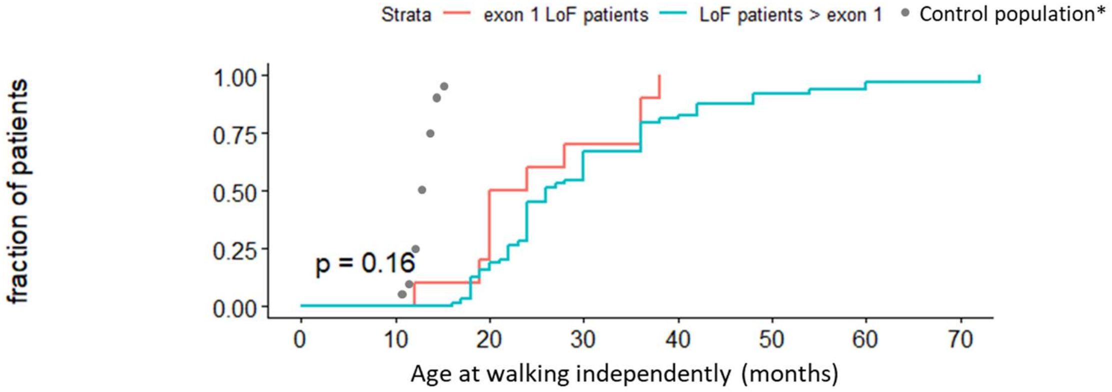

Supplementary Figure 4C) Cumulative distribution: first words, *n*=62

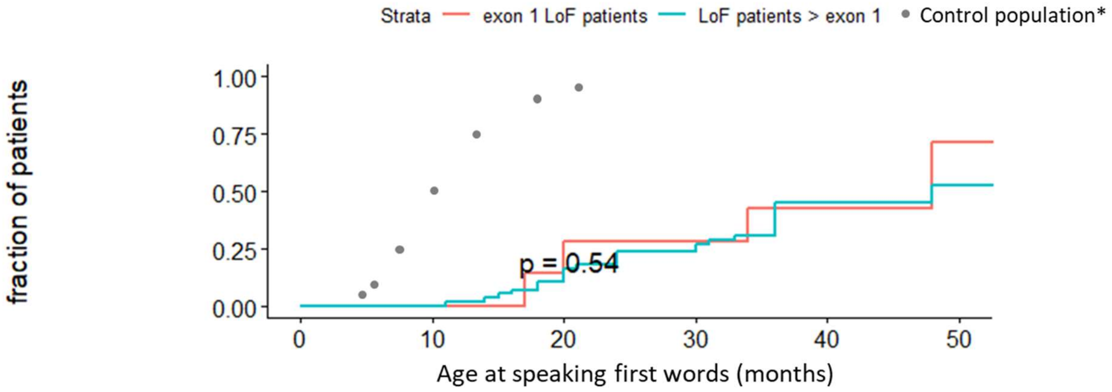

Supplementary Figure 4D) Cumulative distribution: toilet trained stool, *n*=34

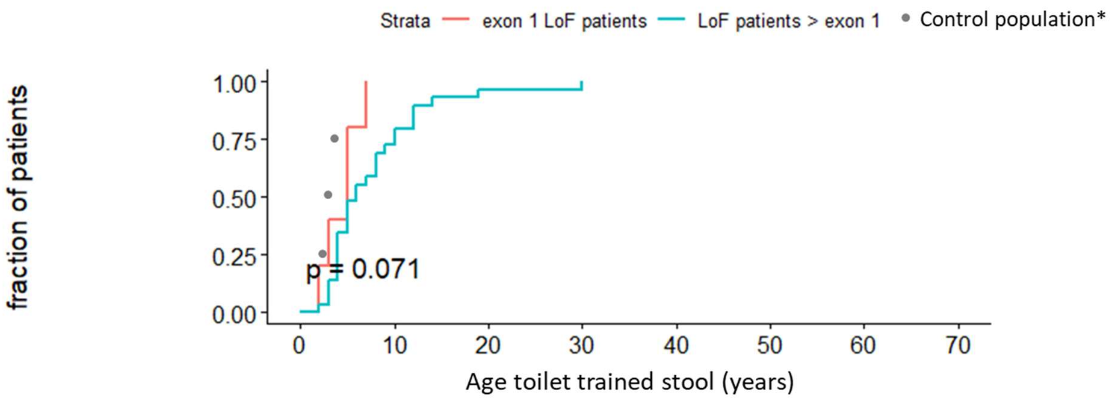

Supplementary Figure 4E) Kaplan-Meier: seizures, *n*=49

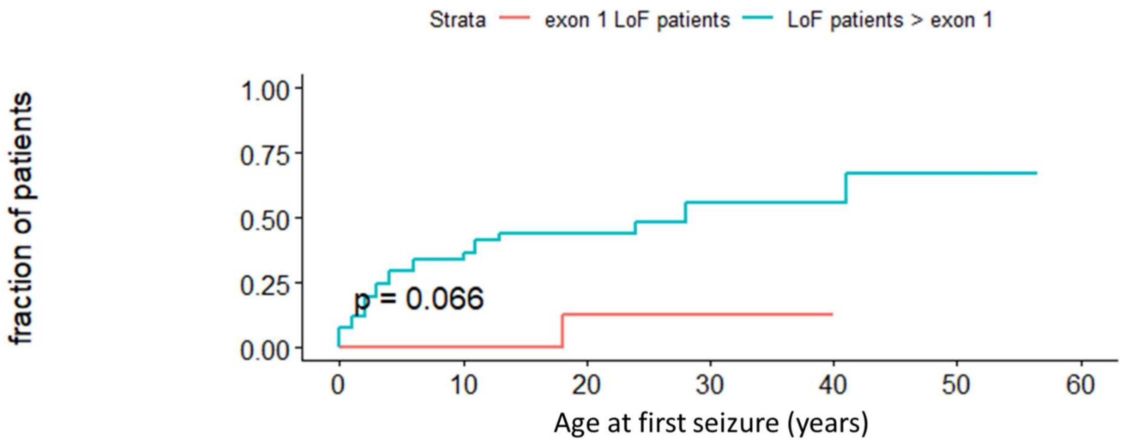

Supplementary Figure 4F) Kaplan-Meier: diabetes mellitus, type II, *n*=53

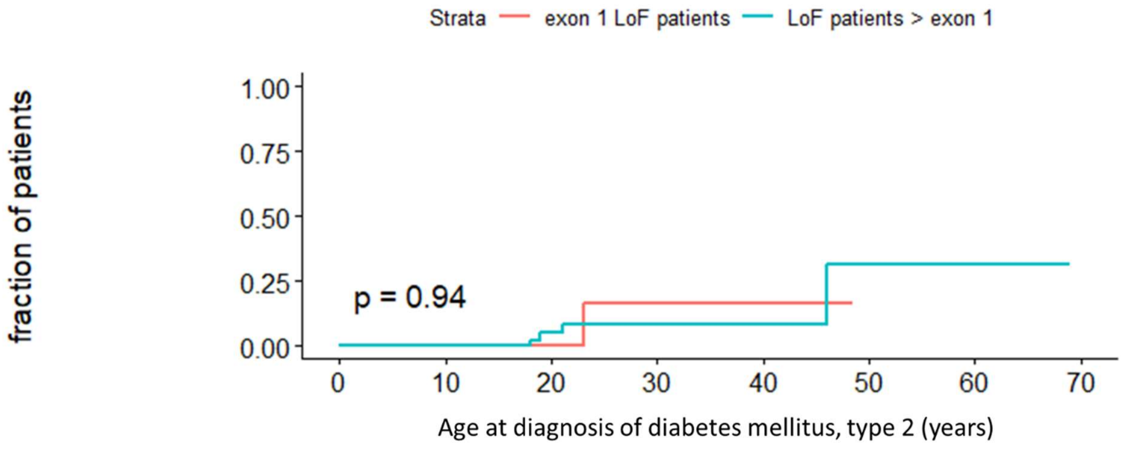

Supplementary Figure 4G) Kaplan-Meier: hypothyroidism, n=51

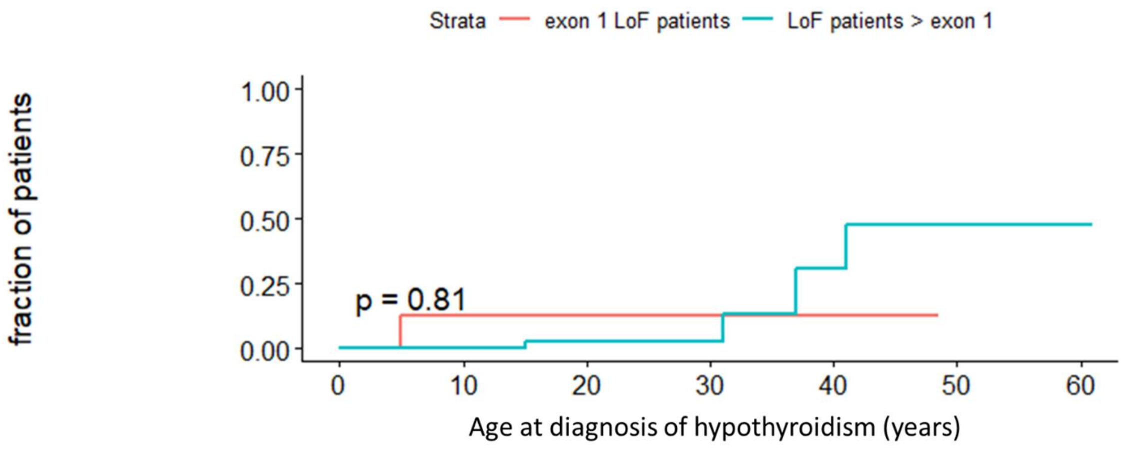

Supplementary Figure 4H) Kaplan-Meier: nephrolithiasis, n=38

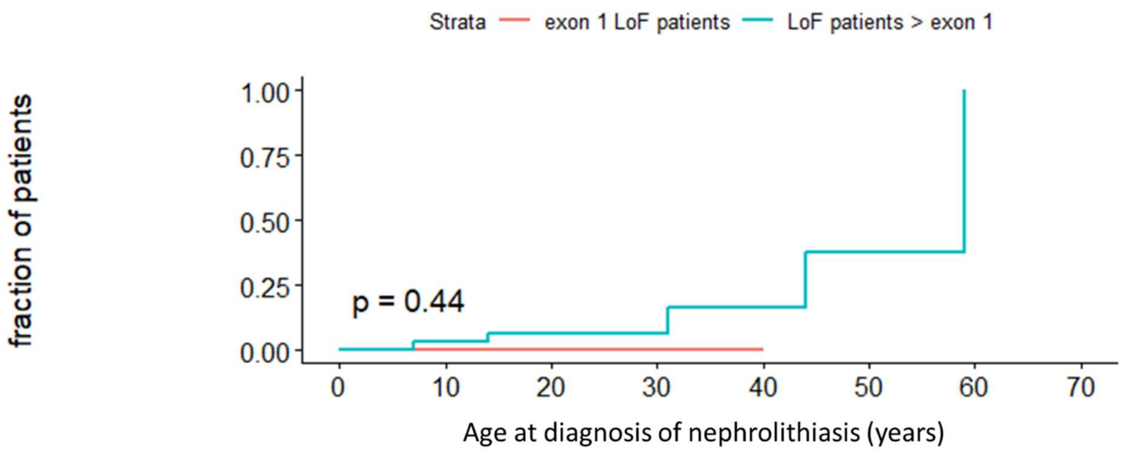

Supplementary Figure 4I) Kaplan-Meier: patella luxation, *n*=42

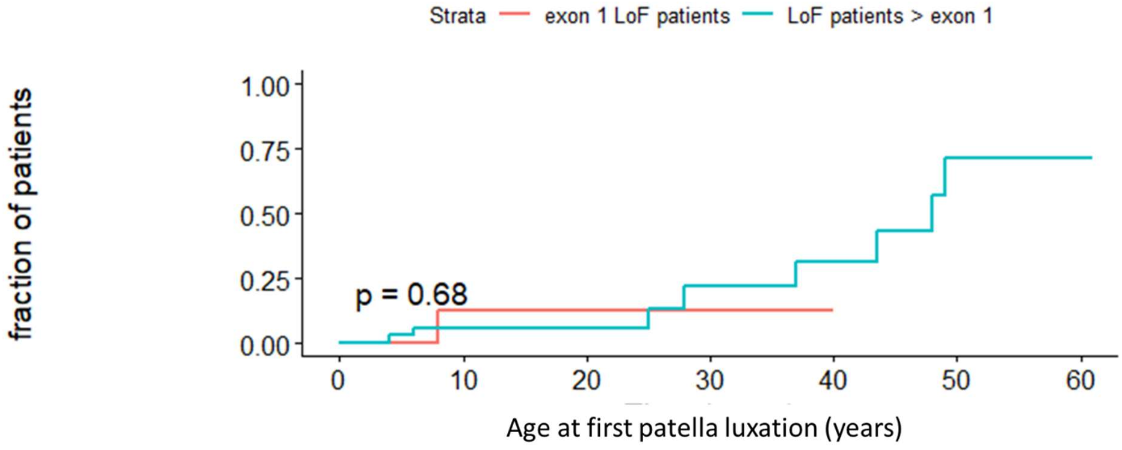

Supplementary Figure 4J) Kaplan-Meier: loss of skill, *n*=52

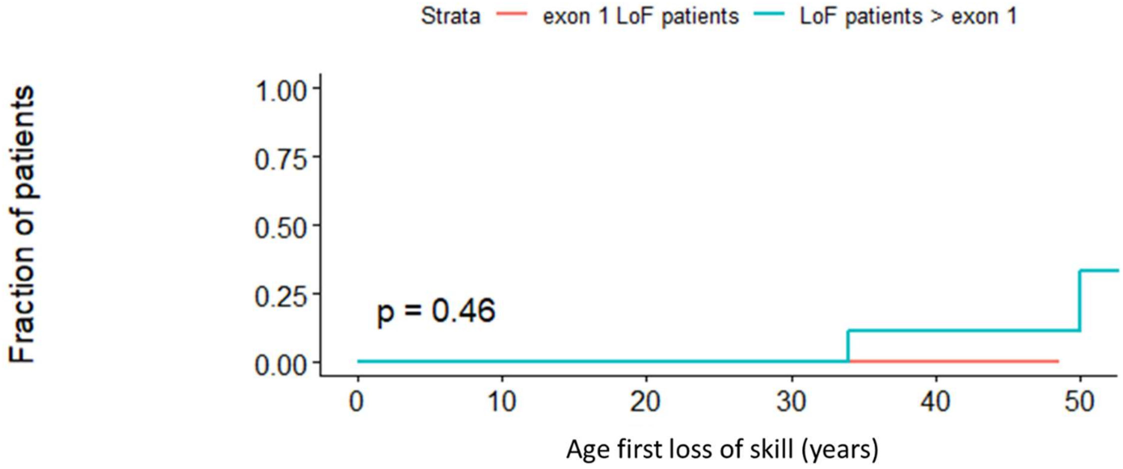

Supplementary Table 4: PhenoScore analyses – all patients

Analysis	Number of photos	Brier	AUC	*p*-value
analysis_1_ARID1B 18+ vs controls	48	0.09	0.94	<0.01
analysis_2_0.0_4.5 vs controls	27	0.12	0.9	<0.01
analysis_2_4.5_10.5 vs controls	26	0.12	0.91	<0.01
analysis_2_10.5_17.5 vs controls	26	0.09	0.95	<0.01
analysis_2_17.5_25.5 vs controls	19	0.11	0.92	<0.01
analysis_2_25.5_200.0 vs controls	22	0.12	0.89	<0.01
analysis_3_above_25 vs controls	10	0.24	0.51	0.05
analysis_3_below_11 vs controls	10	0.23	0.54	<0.01
analysis_4_a_exon1 vs controls	8	0.25	0.56	0.65
analysis_4_b_>exon1 vs controls	43	0.11	0.92	<0.01
analysis_4_c_exon1 vs >exon1 matched	7	0.29	0.61	0.84

Supplementary Figure 5: Facial heatmaps of PhenoScore analyses using photos of all patients

Heatmaps are generated using LIME to see which facial areas are most important according to our model, where blue correlates with *ARID1B* patients and red areas correlate with controls.

Supplementary Figure 5A)
Facial heatmap of analysis_1_ARID1B 18+ vs controls

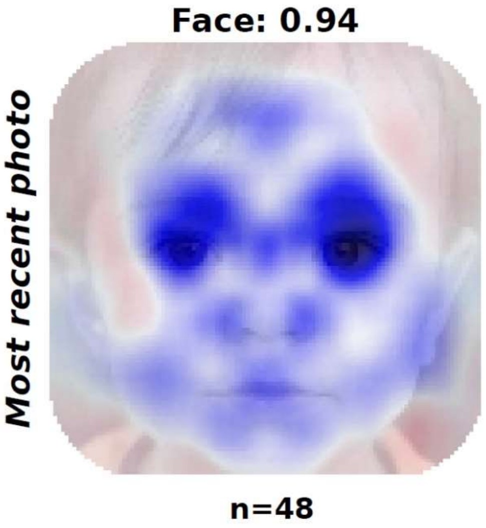

Supplementary Figure 5B)
Facial heatmap of analysis_2_0.0_4.5 vs controls

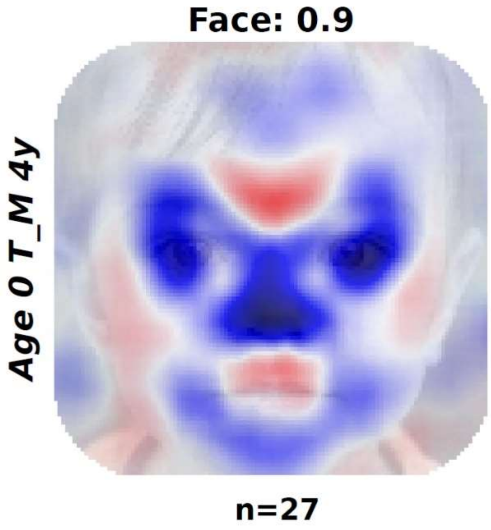

Supplementary Figure 5C)
Facial heatmap of analysis_2_4.5_10.5 vs controls

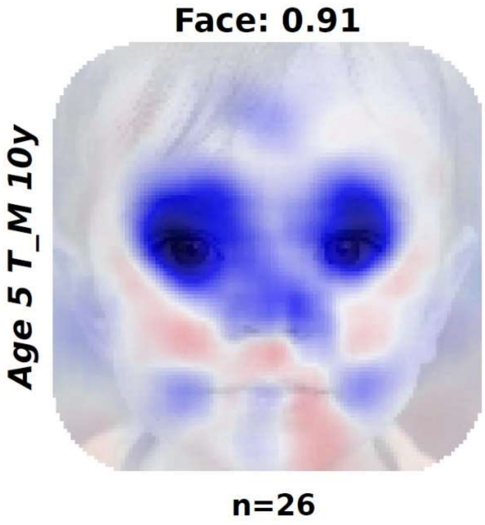

Supplementary Figure 5D)
Facial heatmap of analysis_2_10.5_17.5 vs controls

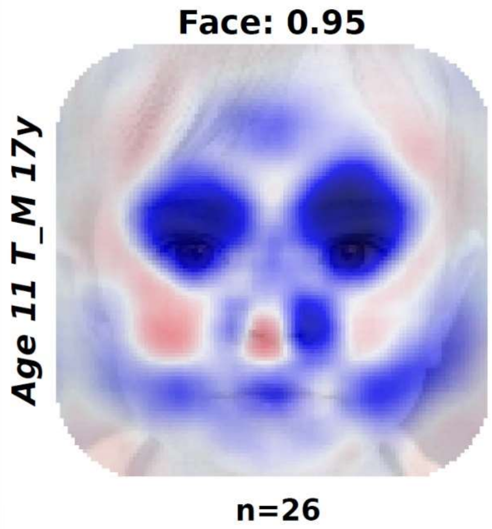

Supplementary Figure 5E)
Facial heatmap of analysis_2_17.5_25.5 vs controls

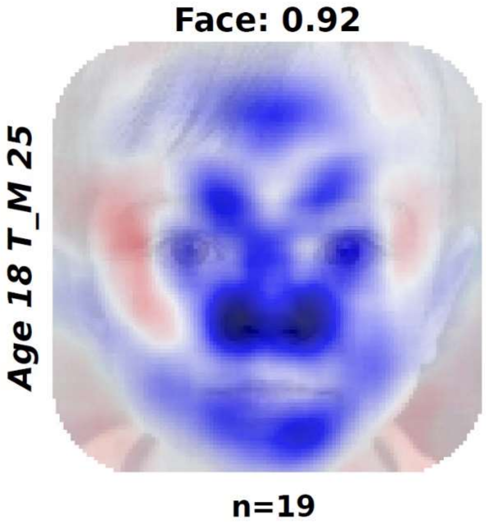

Supplementary Figure 5F)
Facial heatmap of analysis_2_25.5_200 vs controls

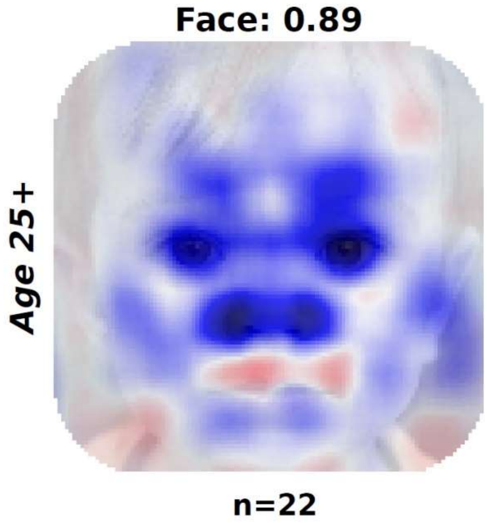

Supplementary Figure 5G)
Facial heatmap of analysis_3_above_25 vs vs controls

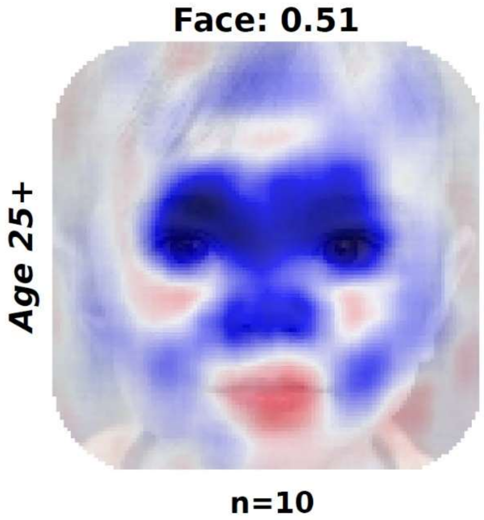

Supplementary Figure 5H)
Facial heatmap of analysis_3_below_11 vs controls

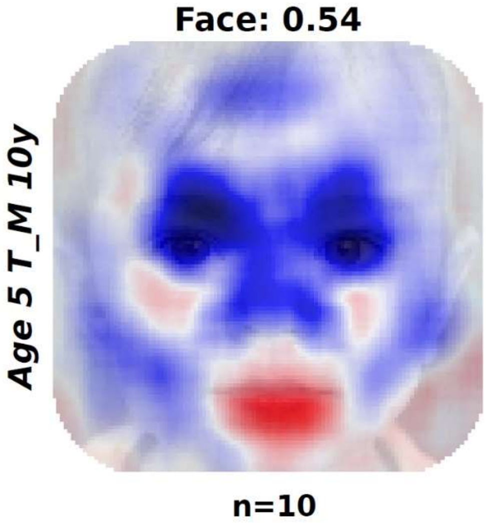

Supplementary Figure 5I)
Facial heatmap of analysis_4_a_exon1 vs controls

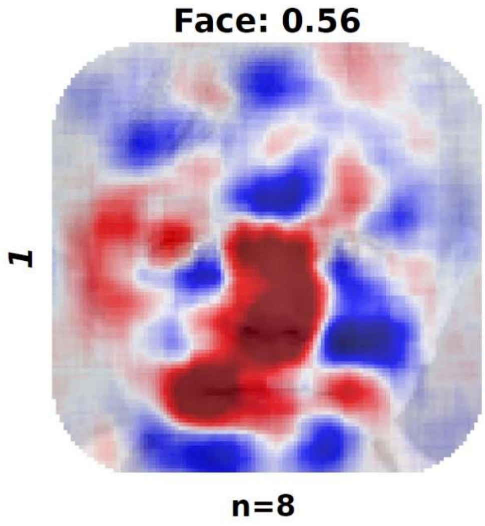

Supplementary Figure 5J)
Facial heatmap of analysis_4_b_>exon1 vs controls

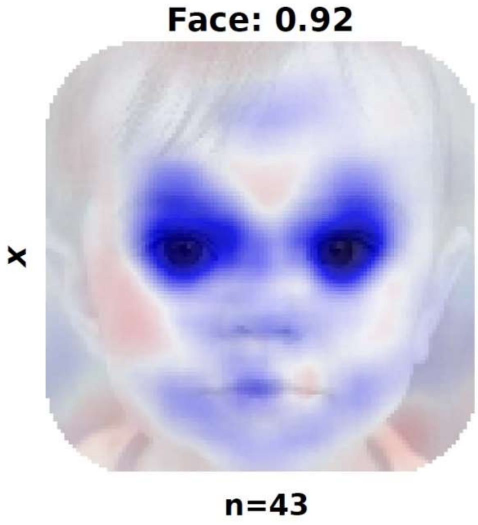

Supplementary Figure 5K)
Facial heatmap of analysis_4_c_exon1 vs >exon1 matched

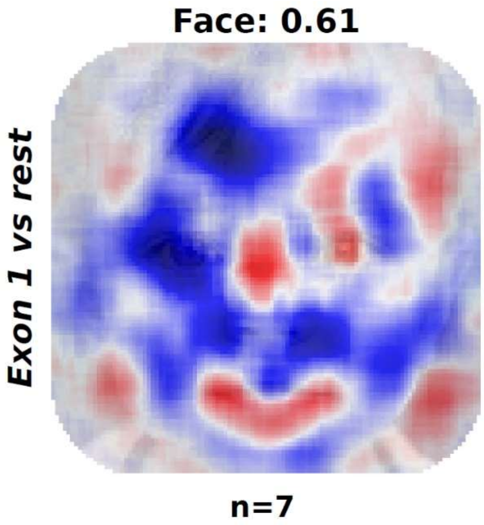

Supplementary Table 5: PhenoScore analyses – mosaic case

Patient ID	Sex	Age (year)	mean_score	score_it_1	score_it_2	score_it_3	score_it_4	score_it_5	classification_phenoscore	analysis_prediction
066	male	0.6	0,81	0,76	0,81	0,88	0,83	0,77	Phenotypic Match	analysis_1_ARID1B 18+ vs controls
066	male	4.0	0,85	0,91	0,87	0,92	0,84	0,7	Phenotypic Match	analysis_1_ARID1B 18+ vs controls
066	male	10.0	0,96	0,95	0,97	0,98	0,94	0,95	Phenotypic Match	analysis_1_ARID1B 18+ vs controls
066	male	18.0	0,71	0,66	0,71	0,74	0,8	0,65	Phenotypic Match	analysis_1_ARID1B 18+ vs controls
066	male	40.0	0,72	0,77	0,81	0,82	0,81	0,39	Phenotypic Match	analysis_1_ARID1B 18+ vs controls
066	male	0.6	0,94	0,98	0,94	0,93	0,96	0,9	Phenotypic Match	analysis_2_0.0_4 vs controls
066	male	4.0	0,95	0,97	0,98	0,93	0,97	0,9	Phenotypic Match	analysis_2_0.0_4 vs controls
066	male	10.0	0,82	0,86	0,83	0,83	0,79	0,8	Phenotypic Match	analysis_2_0.0_4 vs controls
066	male	18.0	0,72	0,8	0,85	0,66	0,86	0,46	Phenotypic Match	analysis_2_0.0_4 vs controls
066	male	40.0	0,61	0,58	0,69	0,57	0,69	0,51	VUS	analysis_2_0.0_4 vs controls
066	male	0.6	0,91	0,91	0,9	0,91	0,93	0,9	Phenotypic Match	analysis_2_5_10 vs controls
066	male	4.0	0,94	0,91	0,97	0,96	0,94	0,93	Phenotypic Match	analysis_2_5_10 vs controls
066	male	10.0	0,94	0,85	0,96	0,98	0,99	0,89	Phenotypic Match	analysis_2_5_10 vs controls
066	male	18.0	0,74	0,55	0,75	0,88	0,97	0,57	Phenotypic Match	analysis_2_5_10 vs controls
066	male	40.0	0,53	0,37	0,4	0,69	0,8	0,39	VUS	analysis_2_5_10 vs controls
066	male	0.6	0,69	0,69	0,67	0,89	0,73	0,49	VUS	analysis_2_11_17 vs controls
066	male	4.0	0,74	0,69	0,8	0,89	0,75	0,56	Phenotypic Match	analysis_2_11_17 vs controls
066	male	10.0	0,94	0,95	0,93	0,98	0,88	0,93	Phenotypic Match	analysis_2_11_17 vs controls
066	male	18.0	0,83	0,92	0,89	0,96	0,68	0,72	Phenotypic Match	analysis_2_11_17 vs controls
066	male	40.0	0,6	0,71	0,54	0,84	0,31	0,57	VUS	analysis_2_11_17 vs controls
066	male	0.6	0,7	0,44	0,91	0,55	0,95	0,63	VUS	analysis_2_18_25 vs controls
066	male	4.0	0,86	0,9	0,86	0,74	0,96	0,84	Phenotypic Match	analysis_2_18_25 vs controls
066	male	10.0	0,92	0,86	0,99	0,92	0,96	0,86	Phenotypic Match	analysis_2_18_25 vs controls
066	male	18.0	0,93	0,93	0,94	0,86	0,96	0,94	Phenotypic Match	analysis_2_18_25 vs controls
066	male	40.0	0,7	0,67	0,72	0,71	0,72	0,69	Phenotypic Match	analysis_2_18_25 vs controls
066	male	0.6	0,72	0,75	0,71	0,66	0,68	0,82	Phenotypic Match	analysis_2_25+ vs controls
066	male	4.0	0,78	0,74	0,78	0,77	0,74	0,86	Phenotypic Match	analysis_2_25+ vs controls
066	male	10.0	0,78	0,76	0,77	0,78	0,71	0,85	Phenotypic Match	analysis_2_25+ vs controls
066	male	18.0	0,57	0,52	0,55	0,39	0,62	0,74	VUS	analysis_2_25+ vs controls
066	male	40.0	0,75	0,8	0,79	0,57	0,8	0,79	Phenotypic Match	analysis_2_25+ vs controls

Supplementary Figure 6: A) Histogram of age included patients B) Histogram of age reported patient cohort in *van der Sluijs et al.* 2018[1]

A) Histogram of age included patients this paper

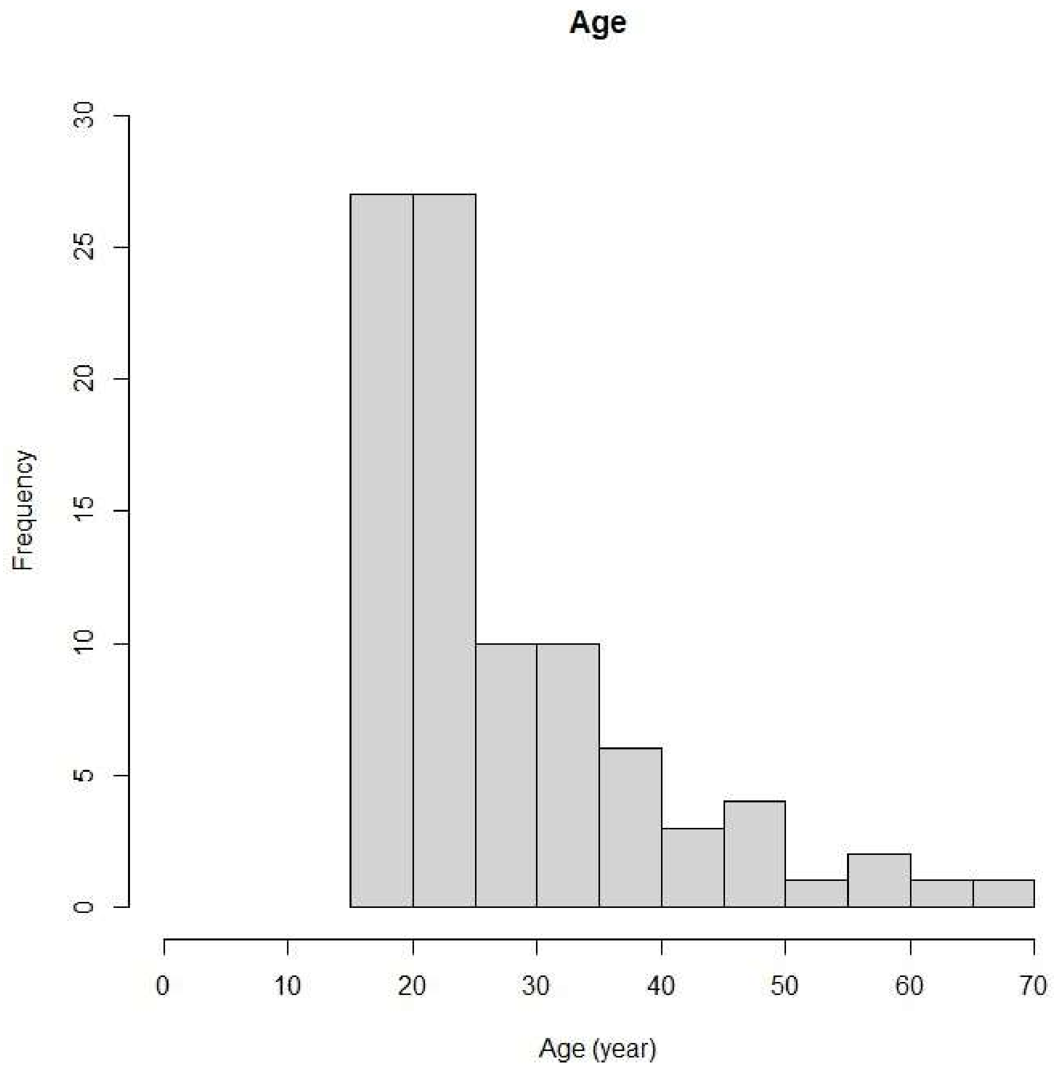

B) Histogram of age reported patient cohort *van der Sluijs et al.* 2018[1]

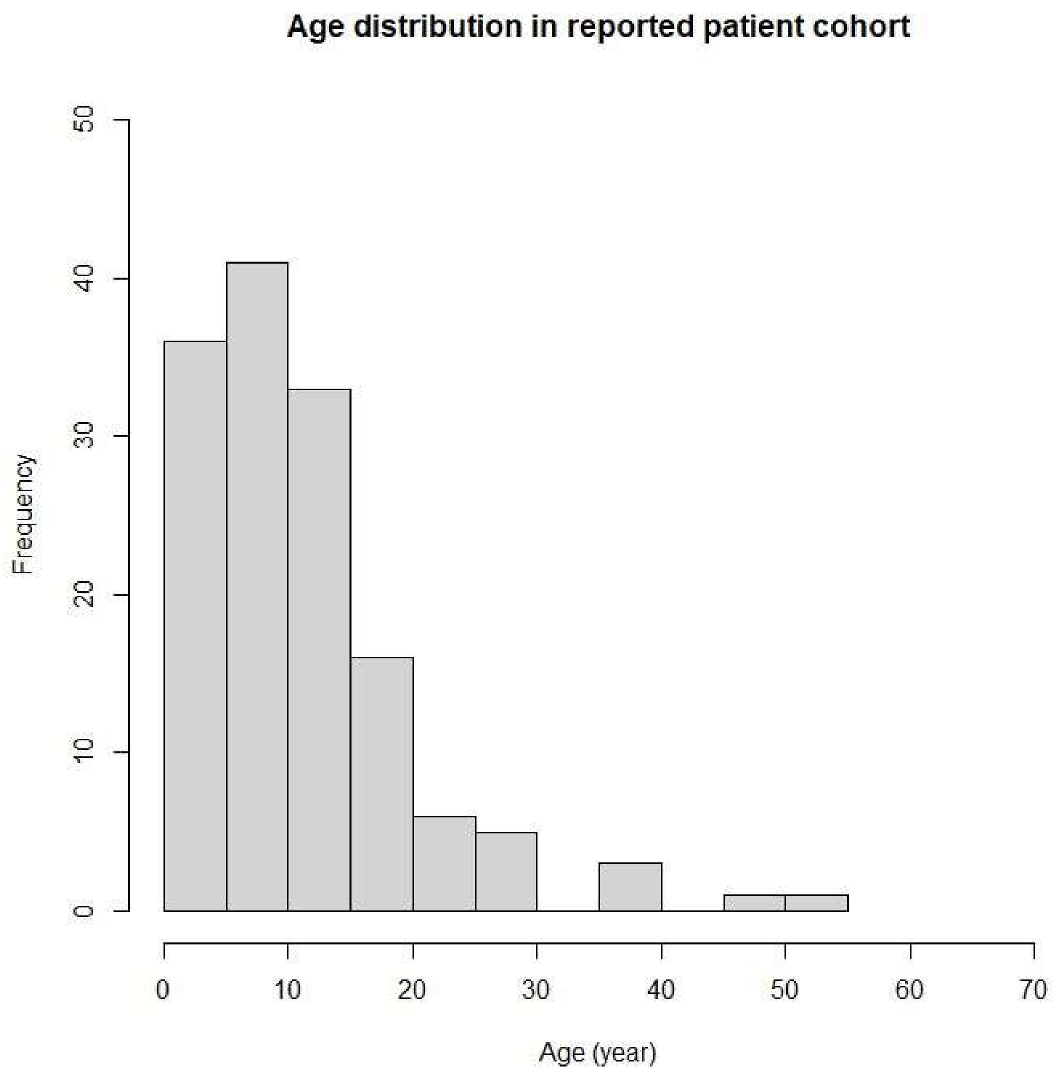

References:

1. van der Sluijs PJ, Jansen S, Vergano SA, et al. The ARID1B spectrum in 143 patients: from nonsyndromic intellectual disability to Coffin–Siris syndrome. *Genetics in Medicine*. 2018/10/22 2018;doi:10.1038/s41436-018-0330-z
2. Sheldrick RC, Schlichting LE, Berger B, et al. Establishing New Norms for Developmental Milestones. *Pediatrics*. Dec 2019;144(6)doi:10.1542/peds.2019-0374
3. Schum TR, Kolb TM, McAuliffe TL, Simms MD, Underhill RL, Lewis M. Sequential acquisition of toilet-training skills: a descriptive study of gender and age differences in normal children. *Pediatrics*. Mar 2002;109(3):E48. doi:10.1542/peds.109.3.e48

Supplementary Table 6: Clinical characteristics of *ARID1B* patients compared with cohort from van der Sluijs *et al.* 2018

	Reported *ARID1B* cohort			18+ (LoF)			LoF patients exon 1			LoF patients >exon 1			Mosaic patients		
Clinical features +	*n* = 143	pt affected	%	*n* = 85	pt affected	%	*n = 16*	pt affected	%	*n = 69*	pt affected	%	*n = 2*	pt affected	%
Sex (female)	143	69	48%	85	52	61%	16	9	56,3%	69	43	62%	2	1	50%
Growth parameters & development															
Gestational age, weeks (mean; SD)	133	39,0	210%	73	39,0	224%	11	38,1	2,8	62	39,1	213%	1	40	-
Birthweight (<-2 SDS)	129	7	5%	53	6	11%	7	0	0,0%	46	6	13%	0	0	-
Height at birth (<-2 SDS)	43	4	9%	16	5	31%	2	0	0,0%	14	5	36%	0	0	-
OFC at birth (<-2 SDS)	51	2	4%	18	1	6%	3	0	0,0%	15	1	7%	0	0	-
Age last measurements, years (n; median; min-max)	143	10	(0-51)	83	22,0	(4,2-69)	16	22,1	(11,8-40)	67	22,0	(4,2-69)	2	36,0	(35-37)
Weight (<-2 SDS)	92	6	7%	55	0	0%	6	0	0,0%	49	0	0%	0	0	-
Lenght (<-2 SDS)	122	37	30%	69	37	54%	9	6	66,7%	60	31	52%	2	1	50%
OFC (<-2 SDS)	105	3	3%	55	3	5%	9	0	0,0%	46	3	7%	1	0	0%
Motor skills gross, delayed	103	102	99%	78	59	76%	14	9	64,3%	64	50	78%	2	0	0%
Motor skills fine, delayed	100	95	95%	78	51	65%	14	6	42,9%	64	45	70%	2	0	0%
Speech, delayed	131	86	66%	81	79	98%	15	14	93,3%	66	65	98%	2	0	0%
Obstructive sleep apnea++	70	6	9%	85	1	1%	16	0	0,0%	69	1	1%	2	0	0%
Laryngomalacia++	90	18	20%	53	5	9%	10	1	10,0%	43	4	9%	2	0	0%
Feeding difficulties	121	84	69%	81	53	65%	15	6	40,0%	66	47	71%	2	0	0%
Duration of feeding problems	58			42			5			37			0		
Brief		27	47%		17	40%		3	60,0%		14	38%		0	-
Several years		4	7%		16	38%		0	0,0%		16	43%		0	-
Ongoing		27	47%		9	21%		2	40,0%		7	19%		0	-
Recurrent infections	75	43	57%	71	28	39%	12	4	33,3%	59	24	41%	1	0	0%
Upper airway tract		13	17%	71	3	4%			0,0%		3	5%	1	0	0%
Lower airway tract		2	3%	71	2	3%			0,0%		2	3%	1	0	0%
ENT infections		9	12%	71	12	17%			8,3%		11	19%	1	0	0%
Otitis media		11	15%	71	10	14%			16,7%		8	14%	1	0	0%
Urinary tract		2	3%	71	6	8%			8,3%		5	8%	1	0	0%
Neurological features															
IQ (median; (min-max))	35	55,0	(26-114)	32	55	(20-80)	4	58-5	(54-65)	28	55,0	(20-80)	0	-	-
Intellectual disability	127	126	99%	85	79	93%	16	13	81,3%	69	66	96%	2	0	0%
Borderline		4	3%	0	6	7%		3	18,8%		3	4%		1	50%
Mild		36	28%	0	24	28%		6	37,5%		18	26%		0	0%
Mild-moderate		20	16%	-	-	-	-	-	-	-	-	-	-	-	-
Moderate		28	22%	0	36	42%		5	31,3%		31	45%		0	0%
Moderate-severe		21	17%	-	-	-	-	-	-	-	-	-	-	-	-
Severe		17	13%	0	17	20%		2	12,5%		15	22%		0	0%
Hypotonia	116	94	81%	74	57	77%	12	7	58,3%	62	50	81%	2	0	0%
Seizures	142	39	27%	81	38	47%	14	5	35,7%	67	33	49%	2	0	0%
No seizures, but abnormal EEG		8	6%		6	7%		1	7,1%		5	7%		0	0%
Agenesis of the corpus callosum	101	29	29%	58	27	47%	8	4	50,0%	50	23	46%	2	0	0%
Partial/hypoplasia		14	14%		18	31%		3	37,5%		15	30%		0	0%
MRI performed				78	66	85%	15	10	66,7%	63	56	89%	1	1	100%
Vision and hearing impairments															
Vision impaired	109	53	49%	82	68	83%	15	13	86,7%	67	55	82%	2	0	0%
Myopia	102	28	27%	60	47	78%	12	10	83,3%	48	37	77%	0	0	-
Hypermetropia	50	9	18%	53	14	26%	8	0	0,0%	45	14	31%	0	0	-
Hearing loss	122	27	22%	79	22	28%	15	4	26,7%	64	18	28%	2	0	0%
Hearing loss, conductive		8	7%	79	11	14%			13,3%		9	14%	2	0	0%
Eartubes	11	7	64%	56	23	41%	11	4	36,4%	45	19	42%	2	0	0%
Hearing aid	5	4	80%	17	6	35%	3	1	33,3%	14	5	36%	0	0	-
Musculoskeletal anomalies															
Scoliosis	123	32	26%	82	25	30%	15	4	26,7%	67	21	31%	2	1	50%
Pectus, excavatum	104	14	13%	80	2	3%	15	0	0,0%	65	2	3%	1	0	0%
Primary dentition, delayed	65	29	45%	47	11	23%	9	1	11,1%	38	10	26%	2	0	0%
Permanent dentition, delayed	33	16	48%	47	19	40%	9	2	22,2%	38	17	45%	2	0	0%
Widely spaced teeth	72	30	42%	47	7	15%	9	1	11,1%	38	6	16%	2	0	0%
Joint laxity	88	53	60%	46	23	50%	8	2	25,0%	38	21	55%	2	0	0%
Early arthritis	75	4	5%	33	2	6%	7	0	0,0%	26	2	8%	1	0	0%
Clinodactyly	77	28	36%	65	8	12%	12	2	16,7%	53	6	11%	2	0	0%
Brachydactyly fifth finger	68	21	31%	65	16	25%	12	3	25,0%	53	13	25%	2	0	0%
Small nails	122	67	55%	66	25	38%	13	6	46,2%	53	19	36%	2	0	0%
Which nails, 5th finger and/or toe	106	59	56%	66	19	29%	13	6	46,2%	53	13	25%	2	0	0%
Intestinal															
Inguinal hernia	90	7	8%	53	4	8%	9	1	11,1%	44	3	7%	1	0	0%
Intestinal problems	105	51	49%	73	31	42%	13	4	30,8%	60	27	45%	1	0	0%
Constipation	105	32	30%	73	20	27%	13	1	7,7%	60	19	32%	1	0	0%
Gastroesophageal reflux	105	18	17%	73	8	11%	13	2	15,4%	60	6	10%	1	0	0%
Diarrhea	105	5	5%	73	0	0%	13	0	0,0%	60	0	0%	1	0	0%
Pyloric Stenosis	105	3	3%	73	0	0%	13		0,0%	60	0	0%	1	0	0%
Umbilical hernia	105	5	5%	73	3	4%	13	2	15,4%	60	1	2%	1	0	0%
Cardiac & urogenital anomalies															
Cardiac anomalies	113	22	19%	65	9	14%	12	1	8,3%	53	8	15%	1	0	0%
ASD	113	12	11%	65	4	6%	12	0	0,0%	53	4	8%	1	0	0%
VSD	113	6	5%	65	0	0%	12	0	0,0%	53	0	0%	1	0	0%
Aortic valve abnormality	113	3	3%	65	2	3%	12	1	8,3%	53	1	2%	1	0	0%
Mitralis insufficiency	113	3	3%	65	1	2%	12	0	0,0%	53	1	2%	1	0	0%
Renal anomalies	95	12	13%	42	18	43%	6	0	0,0%	36	18	50%	0	-	-
Hydronephrotic kidney	95	3	3%	42	4	10%	6	0	0,0%	36	4	11%	0	-	-
Nephrolithiasis	95	3	3%	42	9	21%	6	0	0,0%	36	9	25%	0	-	-
Renal sonography, abnormal	43	11	26%	43	18	42%	6	0	0,0%	37	18	49%	0	-	-
Cryptorchidism	65	36	55%	30	18	60%	6	3	50,0%	24	15	63%	1	0	0%
Endocrinological abnormalities++															
Diabetes mellitus	70	5	7%	54	6	11%	8	1	12,5%	46	5	11%	0	-	-
Type 2 diabetes mellitus	4	3	75%	54	6	11%	8	1	12,5%	46	5	11%	0	-	-
Hypothyroidism	62	12	19%	54	8	15%	8	1	12,5%	46	7	15%	0	-	-
Growth hormone deficiency	50	7	14%	54	1	2%	8	0	0,0%	46	1	2%	0	-	-
Behavioral abnormalities	71	59	83%	80	68	85%	13	10	76,9%	67	58	87%	2	1	50%
Hyperactivity	63	27	43%	75	5	7%	13	0	0,0%	62	5	8%	2	0	0%
High pain threshold++	46	19	41%	53	34	64%	9	4	44,4%	44	30	68%	1	0	0%
Psychiatric disorders															
ADHD	48	16	33%	80	7	9%	13	1	7,7%	67	6	9%	2	1	50%
Autistic traits	77	44	57%	80	21	26%	13	2	15,4%	67	19	28%	2	0	0%
Malignancies	97	1	1%	73	1	1%	12	0	0,0%	61	1	2%	2	0	0%

+ the total number of a feature can differ from the sum of subcategories, because in some cases it was possible to answer with more than 1 option or to report the existence of a feature without specifying.

++ in van der Sluijs *et al.* 2018 data regarding these features was collected seperately through e-mail after the first analyses.

Abbreviations

SDS: Standard Deviation Score
OFC: OccipitoFrontal Circumference
SD: Standard Deviation
ENT: Ear Nose Throat
EEG: ElectroEncephaloGraphy
ASD: Atrial Septal Defect
VSD: Ventricular Septal Defect
ADHD: Attention Deficit Hyperactivity D
LoF: Loss of Function variants
F: Fisher's exact
Chi: Chi-square
T: T-test
A: ANOVA
KW: Kruskal-Wallis
MW: Mann-Whitney U

Supplementary Table 7: Clinical characteristics of *ARID1B* patients and the cohort from van der Sluijs *et al.* 2018 with a statistical comparison

	Reported *ARID1B* cohort			vs 18+ (LoF)		18+ (LoF)					LoF patients exon 1			LoF patients >exon 1			Mosaic patients		
Clinical features +	*n* = 143	pt affected	%	*p-value**	*Test**	*n* = 85	pt affected	%	*p-value**	*Test**	*n = 16*	pt affected	%	*n = 69*	pt affected	%	*n = 2*	pt affected	%
Weight (<-2 SDS)	92	6	7%	0,08	F	55	0	0%	-	-	6	0	0%	49	0	0%	0	0	-
Lenght (<-2 SDS)	122	37	30%	0,00	Chi	69	37	54%	0,49	F	9	6	67%	60	31	52%	2	1	50%
OFC (<-2 SDS)	105	3	3%	0,41	F	55	3	5%	1,00	F	9	0	0%	46	3	7%	1	0	0%
Motor delay	103	102	99%	0,04	F	78	72	92%	0,01	F	14	10	71%	64	62	97%	2	0	0%
Feeding difficulties	121	84	69%	0,55	Chi	81	53	65%	0,03	Chi	15	6	40%	66	47	71%	2	0	0%
Ongoing		27	47%	0,01	Chi		9	21%				2	40%		7	19%		0	-
Recurrent infections	75	43	57%	0,03	Chi	71	28	39%	0,75	F	12	4	33%	59	24	41%	1	0	0%
Intellectual disability	127	126	99%	0,00	F	85	79	93%	0,08	F	16	13	81%	69	66	96%	2	0	0%
Seizures	142	39	27%	0,00	Chi	81	38	47%	0,67	F	14	5	36%	67	33	49%	2	0	0%
Vision impaired	109	53	49%	<0.01	Chi	82	68	83%	1,00	F	15	13	87%	67	55	82%	2	0	0%
Myopia	102	28	27%	<0.01	Chi	60	47	78%	1,00	F	12	10	83%	48	37	77%	0	0	-
Hypermetropia	50	9	18%	0,31	Chi	53	14	26%	0,09	F	8	0	0%	45	14	31%	0	0	-
Hearing loss	122	27	22%	0,36	Chi	79	22	28%	1,00	F	15	4	27%	64	18	28%	2	0	0%
Eartubes	11	7	64%	0,20	F	56	23	41%	1,00	F	11	4	36%	45	19	42%	2	0	0%
Hearing aid	5	4	80%	0,14	F	17	6	35%	1,00	F	3	1	33%	14	5	36%	0	0	-
Scoliosis	123	32	26%	0,48	F	82	25	30%	1,00	F	15	4	27%	67	21	31%	2	1	50%
Abnormal dentition	83	37	45%	0,01	Chi	36	25	69%	1,00	F	4	3	75%	32	22	69%	2	1	50%
Brachydactyly fifth finger	68	21	31%	0,42	Chi	65	16	25%	1,00	F	12	3	25%	53	13	25%	2	0	0%
Small nails	122	67	55%	0,03	Chi	66	25	38%	1,00	Chi	13	6	46%	53	19	36%	2	0	0%
Which nails, 5th finger and/or toe	106	59	56%	<0.01	Chi	66	19	29%	0,17	Chi	13	6	46%	53	13	25%	2	0	0%
Intestinal problems	105	51	49%	0,42	Chi	73	31	42%	0,54	F	13	4	31%	60	27	45%	1	0	0%
Cardiac anomalies	113	22	19%	0,34	Chi	65	9	14%	1,00	F	12	1	8%	53	8	15%	1	0	0%
Renal anomalies	95	12	13%	<0.01	Chi	42	18	43%	0,01	F	6	0	0%	36	18	50%	0	-	-
Cryptorchidism	65	36	55%	0,67	Chi	30	18	60%	0,66	F	6	3	50%	24	15	63%	1	0	0%
Diabetes mellitus	70	5	7%	0,44	Chi	54	6	11%	1,00	F	8	1	13%	46	5	11%	0	-	-
Hypothyroidism	62	12	19%	0,52	Chi	54	8	15%	1,00	F	8	1	13%	46	7	15%	0	-	-
Growth hormone deficiency	50	7	14%	0,03	F	54	1	2%	1,00	F	8	0	0%	46	1	2%	0	-	-
Behavioral abnormalities	71	59	83%	0,75	Chi	80	68	85%	0,40	F	13	10	77%	67	58	87%	2	1	50%
Hyperactivity	63	27	43%	<0,01	Chi	75	5	7%	0,59	F	13	0	0%	62	5	8%	2	0	0%
High pain threshold	46	19	41%	0,02	Chi	53	34	64%	0,26	F	9	4	44%	44	30	68%	1	0	0%
ADHD	48	16	33%	0,00	Chi	80	7	9%	1,00	F	13	1	8%	67	6	9%	2	1	50%
Autistic traits	77	44	57%	<0,01	Chi	80	21	26%	0,50	F	13	2	15%	67	19	28%	2	0	0%
Malignancies	97	1	1%	1,00	F	73	1	1%	1,00	F	12	0	0%	61	1	2%	2	0	0%

+ the total number of a feature can differ from the sum of subcategories, because in some cases it was possible to answer with more than 1 option or to report the existence of a feature without specifying.

* Groups compared are reported patients (*n* =143) versus non-mosaic patients with a pathogenic variant in *ARID1B* (*n* =85) from this paper.

Abbreviations

SDS: Standard Deviation Score
OFC: OccipitoFrontal Circumference
SD: Standard Deviation
ENT: Ear Nose Throat
EEG: ElectroEncephaloGraphy
ASD: Atrial Septal Defect
VSD: Ventricular Septal Defect
ADHD: Attention Deficit Hyperactivity Disorder
LoF: Loss of Function variants
F: Fisher's exact
Chi: Chi-square
T: T-test
A: ANOVA
KW: Kruskal-Wallis
MW: Mann-Whitney U
